# An evaluation of the psychometric properties of the Australian Collaborative Practice Assessment Tool

**DOI:** 10.1371/journal.pone.0302834

**Published:** 2024-05-09

**Authors:** Bau Dilam Ardyansyah, Reinie Cordier, Margo Brewer, Dave Parsons

**Affiliations:** 1 Curtin School of Allied Health, Faculty of Health Sciences, Curtin University, Perth, Australia; 2 Department of Medical Education, Faculty of Medicine, Hasanuddin University, Makassar, South Sulawesi, Indonesia; 3 Department of Social Work, Education and Community Wellbeing, Faculty of Health and Life Sciences, Northumbria University, Newcastle upon the Tyne, United Kingdom; 4 Department of Health and Rehabilitation Sciences, Faculty of Health Sciences, University of Cape Town, Cape Town, South Africa; Guangxi Normal University, CHINA

## Abstract

**Objectives:**

This study aimed to validate the Collaborative Practice Assessment Tool (CPAT) in the Australian setting and provide a quality instrument in terms of psychometric properties that can be used to measure interprofessional outcomes for both healthcare practitioners and students. The outcomes evaluated include the capacity to work in an interprofessional team, good interprofessional communication skills, leadership skills, ensuring clear division of tasks and roles in a team, effective conflict management, and being actively involved with patients and their families/communities in care.

**Methods:**

The COSMIN (**CO**nsensus-based **S**tandards for the selection of health **M**easurement **IN**struments) taxonomy and standards were used as guides for evaluating the psychometric properties of the Australian CPAT, which include evaluations regarding instrument development requirements of sample target and size, content validity, internal structure (structural validity, internal consistency reliability and measurement invariance), and hypotheses testing. CPAT Australia was developed through two stages involving pilot studies and a validation study, both of which included healthcare practitioners and students as participants. A pilot study examined content validity regarding item relevance, item comprehensibility, and instrument comprehensiveness. The validation study was carried out to assess the internal structure of CPAT Australia for aspects of structural validity, internal consistency reliabilities, and configural, metric and scalar measurement invariance. The structural validity was explored using the following three steps: exploratory, confirmatory, and multi-group factor analysis. Construct validity was evaluated to confirm direct and indirect paths of assumptions based on a previously validated model. Data collected between August 2021 and May 2022.

**Results:**

The content validity evaluation confirmed that all items were relevant, understandable and comprehensive for measuring interprofessional collaborative care in Australia. Three hundred ninety-nine participants contributed to the validation study (*n*=152 practitioners; *n*=247 students). The original instrument model of 8-Factor 56-Item was improved in the Australian CPAT. Two items, Item 27 (Physicians assume the ultimate responsibility) and Item 49 (Final decision rest with the physician), were consistently rejected and therefore discarded. The internal structure of the 7-Factor 54-Item solution was confirmed as a suitable model with fit indices meeting COSMIN standards for a good model in practitioner and student cohorts. Configural, metric and scalar invariances were confirmed, indicating the invariance of the instruments when used for the practitioner and student cohorts. The construct validity evaluation indicated that 81.3% of direct and indirect assumptions were accepted, fulfilling the COSMIN requirement of >75% of proposed assumptions being accepted.

**Conclusion:**

The Australian CPAT with a 7-factor 54-item solution was confirmed as a quality measure for assessing interprofessional education and collaborative practice for both healthcare practitioners and students in Australia with robust psychometric properties.

## Introduction

Working in the same healthcare context, such as the same ward in a hospital, does not constitute an interprofessional education and collaborative practice activity as this group of health professionals may operate within a multiprofessional team framework. The terms multiprofessional and interprofessional practice are sometimes incorrectly used as though they are synonymous. Within a multiprofessional context, health professionals learn alongside each other and do not necessarily learn and work collaboratively or pursue common goals [1]. Conversely, interprofessional education or collaborative practice offers more than parallel learning within an interprofessional context. The nature of interprofessional activities requires health professionals to engage in collective actions to learn with, from, and about each other to pursue collaborative goals. The World Health Organisation defines Interprofessional education as occurring when, “…two or more professions learn about, from and with each other to enable effective collaboration and improve health outcomes” [2, p. 13], and collaborative practice as occurring when, “…multiple health workers from different professional backgrounds provide comprehensive services by working with patients, their families, carers and communities to deliver the highest quality of care across settings” [2, p. 13].

Interprofessional education and collaborative practice have been reported to positively impact patient satisfaction and reduce the duration of hospitalisation, the number of hospital visits, malpractice acts, and health-related costs [2]. However, their implementation in educational settings is considerably challenged by leadership support, timetable restrictions, limited opportunity for collaborative interaction between the students, their mentors and patients, and a lack of resources for appropriate interprofessional learning [3–8]. Australian health education institutions and practice settings are not new to interprofessional education and collaborative practice. The Australian Health Practitioner Regulation Agency (AHPRA), a national agency for accreditation and regulation overseeing 28 health professional bodies, has released a statement of intent regarding interprofessional collaborative practice (IPCP), and interprofessional education has become embedded at the Australian higher education level [9–11]. Several instruments have been used in Australian universities to measure interprofessional related outcomes: the Interprofessional Socialization and Valuing Scale (ISVS), the Readiness for Interprofessional Learning Scale (RIPLS), the University of West England (UWE) instruments, and Curtin University’s Interprofessional Capability Assessment Tool (ICAT) [12]. However, to date, no interprofessional outcomes measure has been validated for either students or practitioners in Australia. Instrument validation is critical to instrument quality control, which, in turn, determines data reproducibility, accuracy of findings, and generalisability [13, 14].

In cross-population studies involving healthcare practitioners and students, as in this current study, it is likely that the instrument will not produce equivalent responses due to differences in factors such as age, length of service or experience, professional backgrounds and practice settings. However, outcome measures for practitioners and students are needed to support greater integration between interprofessional education (at the students’ level) and collaborative practice in the workplaces (at the practitioners’ level). Therefore, it is crucial to investigate the equivalence of statistical models to determine whether the responses of these two populations differ by more than chance. This is an essential process because instrument validation must be conducted in the intended context and with the user population to ensure the adopted items adequately reflect the measure as intended in the original version [13, 15]. Instruments validated for health practitioners are not recommended for use by health students, as practitioners may respond differently to items compared to students, and vice versa [16]. Unfortunately, few instruments are available to assess outcomes at both levels. Outcomes evaluated in the instrument should apply to both cohorts, for example, the capacity to work in an interprofessional team, good interprofessional communication skills, leadership skills, ensuring clear division of tasks and roles in a team, effective conflict management, and being actively involved with patients and their families/communities in care. The Interprofessional Socialization and Valuing Scale (ISVS)-21 is among the few invariant measures for this purpose [17, 18]. However, as opposed to the ISVS-21, the CPAT was chosen in the current study because it is more comprehensive. It contains domains related to patient engagement and community empowerment, constructs not covered in the ISVS-21.

Having an invariant measure between healthcare practitioners and students means that although the two groups may differ in their average levels of agreement with certain factors, the estimated loadings, mean scores and intercepts of items within those factors differ only by chance, thus allowing comparison of the scale scores of both groups [19, 20]. Scores associated with relevant interprofessional outcomes can be compared to identify weaknesses, improvements, and target attainment across the tested groups. The comparable scores enable the identification of the nature of practitioners’ collaborative practice in the workplace and the level of interprofessional development of students in their training.

### The Collaborative Practice Assessment Tool (CPAT)

The Collaborative Practice Assessment Tool (CPAT) is considered a valuable instrument for the Australian context because it covers essential aspects related to interprofessional collaboration, such as team leadership, shared goals, roles clarification, teamwork, and team communication, and contains variables that can be used as patient and community indicators of empowerment in care [21]. The lack of tools to assess organisational or patient care outcomes is an issue related to interprofessional education and collaborative care [22]. The CPAT can provide evidence linking interprofessional education to collaborative practice, including patient involvement and outcomes.

In addition, the CPAT has also been translated and used in many settings worldwide to measure team performance and cross-cultural validation of the instrument has been conducted in Japan [23], Taiwan [24], Indonesia [25], and Singapore [26] and used in many studies in the USA and Canada [27–32]. CPAT is acceptable for use in many different clinical settings, such as primary and community care [29, 33], mental health [23], chronic disease management [28], health professionals postgraduate training [24], patient safety [30], and patient satisfaction [32]. Despite its lengthy scale consisting of 56 items, the CPAT is recommended as the best instrument for measuring interprofessional collaboration for teamwork [34]. All of these studies included health professionals as participants. An American study used CPAT to measure improvements in the health of diabetes patients following an interprofessional collaborative practice program; this research involved both practitioner staff and students as participants [31]. To the best of our knowledge, no study has validated the CPAT for health students.

The original version of the CPAT was designed in Canada in English as a self-assessment measure and consists of 56 items, eight subscales, and three open-ended questions [21]. The subscales are 1) Mission, meaningful purpose, goals (8 items, Cronbach’s *α*=0.88); 2) General relationships (8 items, Cronbach’s *α*=0.89); 3) Team leadership (8 items, Cronbach’s *α*=0.80); 4) General roles responsibilities, autonomy (10 items, Cronbach’s *α*=0.81); 5) Communication and information exchange (6 items, Cronbach’s *α*=0.84); 6) Community linkages and coordination of care (4 items, Cronbach’s *α*=0.76); 7) Decision-making and conflict management (6 items, Cronbach’s *α*=0.67); and 8) Patient involvement (5 items, Cronbach’s *α*=0.87). Six of the items are negatively worded. The scale descriptor was based on a 1 to 7-point Likert scale (1 = Strongly disagree, 2 = Mostly disagree, 3 = Somewhat disagree, 4 = Neither agree nor disagree, 5 = Somewhat agree, 6 = Mostly agree, and 7 = Strongly Agree).

The original CPAT development included two pilot studies involving Canadian healthcare practitioners. Exploratory factor analysis (EFA) was used in the first pilot (*n*=42) to confirm the factorial number, item positioning, and item deletion and Confirmatory factor analysis (CFA) was used in the second pilot analysis (*n*=111). Based on subscale analysis, CFA confirmed that six of the eight subscales met the predefined standards of a ‘good’ fit model based on the normed fit index (NFI), comparative fit index (CFI), Tucker-Lewis index (TLI) and root mean square error of approximation (RMSEA). Two domains, *Communication* and *Patient Involvement*, did not meet these model fit indices. The sample sizes were too small for both factor analysis conducted in the study. The psychometric properties of the CPAT were explored in cross-cultural studies including in Taiwan (*n*=43) [24], Indonesia (*n*=304) [25], and Singapore (*n*=148) [26]. The psychometric properties assessed were mainly related to content validity and internal consistency reliability with Cronbach’s *α*. CPAT subscale analysis was carried out using exploratory factor analysis in the Indonesian study [25] and item-level analysis in the Taiwanese version [24]. The model suggested by the original instrument (8-Factor 56-Item solution) was not tested for a model fit in the three studies.

### Interprofessional practice framework for construct validity

Following COSMIN requirements for construct validity, this study used a causal model for the interprofessional collaboration conceptual framework to estimate the covariance matrix of the population tested [35]. This theoretical framework was chosen because it comprehensively covers relevant aspects of interprofessional collaborative practice and is conceptualised based on validated processes. The causal model for hypotheses testing is presented in the supplementary S1 Fig. Based on this model, there are three levels of constructs: antecedent, mediators, and consequences. The antecedent factors influence interprofessional collaborative practice. Interprofessional collaborative practice mediates the antecedent causal factors and is conceptualised to positively affect the achievement of results, which are the consequences.

Referring to the original CPAT domains, *Communication and information exchange* and *Team leadership* are independent factors that are antecedents in the framework. *Mission, meaning, purpose and goals* and *General roles responsibilities, and autonomy* are mediators. *General relationships*, *Decision-making and conflict management*, *Community linkages and coordination of care*, and *Patient involvement* are independent factors that are consequences of interprofessional collaborative practice. The assumptions tested involved direct and indirect paths, as suggested in this model.

### Objectives

We aimed to provide quality interprofessional education and collaborative practice outcomes measure for Australian healthcare practitioners and students by validating the CPAT with its intended users (healthcare practitioners and students). The COSMIN (**CO**nsensus-based **S**tandards for the selection of health **M**easurement **IN**struments) taxonomy and standards of psychometric properties [13–15] were used to evaluate the psychometric properties of the validated instrument in terms of content validity, internal structure (structural validity, internal consistency reliability, and measurement invariance), and construct validity. This study started with the important step of conducting a pilot study to evaluate content validity, one of COSMIN’s main requirements for item development [13–15]. Content validity checks ensure that the items developed represent the construct being measured in the desired cultural setting. The CPAT has yet to be validated in Australia, so it is important to conduct content validity checks before validating the instrument. In addition to conducting more comprehensive tests of psychometric properties compared to previously reported studies, this paper proposes something new by conductin, 1) an evaluation of the internal structure of the instrument involving measurement invariance, and 2) hypotheses testing for construct validity using previously validated path models [35]. An invariant measure validates the use of the Australian CPAT in practitioner and student equivalently, thus enabling the measurement of outcomes at both levels. The measure is expected to evaluate better integration between interprofessional education and collaborative practice throughout the professional lifespan from pre-qualifying students to experienced health practitioners.

## Materials and methods

### Instrument

The Collaborative Practice Assessment tool (CPAT) is a measure of interprofessional collaborative practice among health practitioners [21]. The original version of the CPAT was designed in English as a self-assessment measure and consists of 56 items, eight subscales, and three open-ended questions [21]. The lead author contacted the developers of the original CPAT to obtain their consent to utilise the instrument in this study.

### Ethics statement

This study gained ethical approval from Curtin University Human Research Ethics Committee (approval number: HRE2021–0274). The survey was circulated between 6 August 2021 and 15 May 2022. Prospective participants were sent an invitation to participate via an online Qualtrics survey [36]. Invitations to practitioners and students were advertised on the official websites and social media accounts of health professional bodies and associations in Australia and via official staff and student communication platforms at university, faculty and study program levels at the researchers’ university. The invitation was provided with information sheets completed with the study details and the consent process. Participation was voluntary, and the responses were anonymous. Each participant was asked to provide their written consent before participating in the study. In addition, as the participants were required to have consented before starting the survey, their consent was assumed based on the survey completion.

### Study procedure

**Fig 1:**
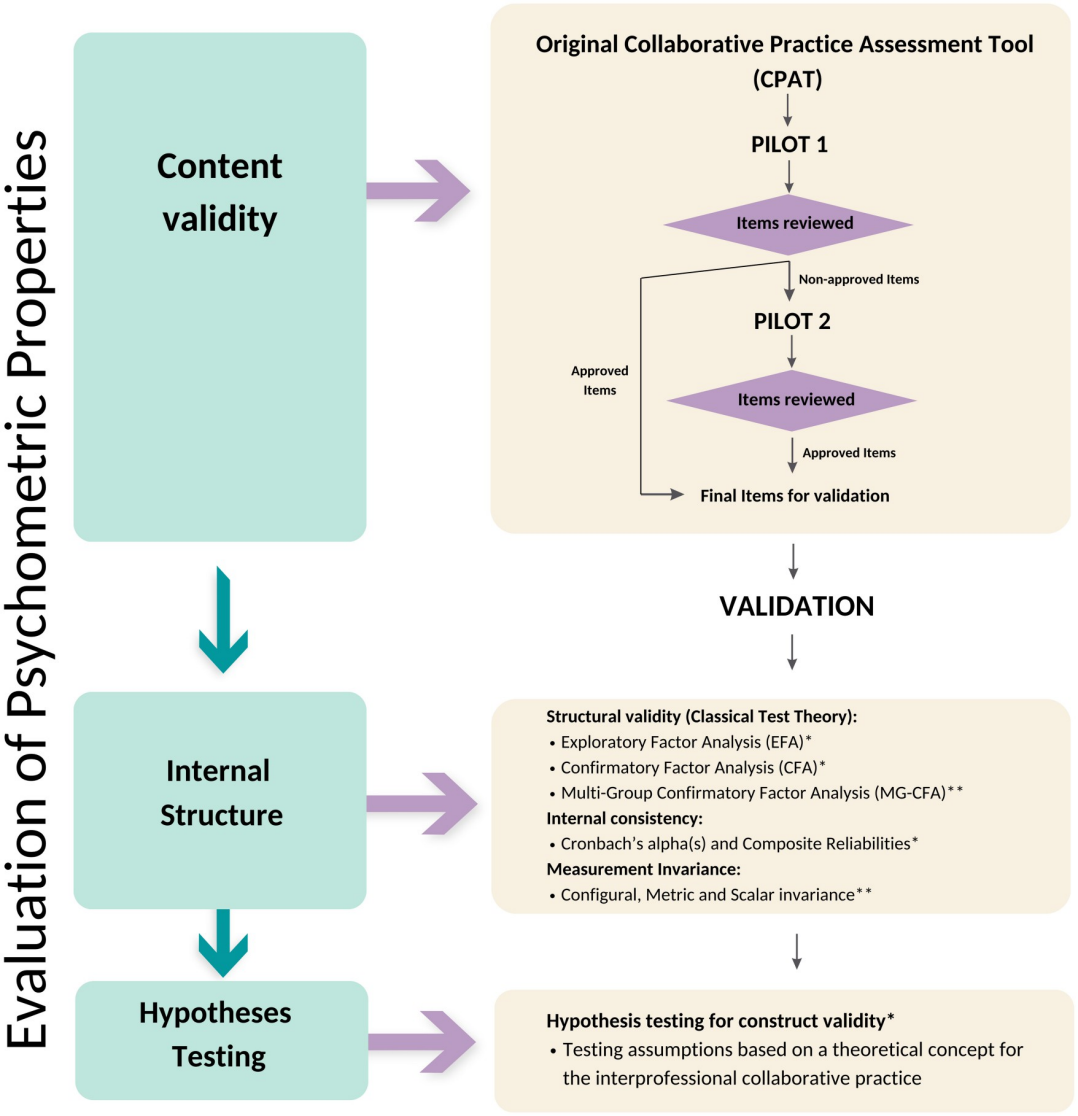
Study procedure.

To evaluate the psychometric properties of the Australian CPAT, we used the COSMIN taxonomy and standards to guide the study procedure. The COSMIN framework for psychometric analysis has been simplified to include only the steps relevant to this study and is presented in Fig 1. Following COSMIN guidelines, the evaluations carried out and the types of psychometric parameters analysed are arranged sequentially. There are three major evaluation themes regarding content validity, internal structure, and hypothesis testing for construct validity.

### Participants

The participants of this study were Australian healthcare practitioners and students selected through purposive sampling to ensure the intended users of the instruments were adequately represented [13–15]. The eligibility criteria for study participation were: 1) Participants needed to be Australian healthcare practitioners and students from any health professional background, and 2) have experience in collaborating within a healthcare team(s) comprised of practitioners or students from different health professions (i.e., an interprofessional team). Because professional role identity occurs at least six months after the start of clinical exposure [37], recruited students and practitioners needed to have at least one year of experience studying or working in an interprofessional team.

#### Pilot study

A total of 31 health practitioners (*n*=22) and students (*n*=9) completed Pilot Study 1. Practitioners were aged between 20 and 59 years (*M*=38.6; *SD*=8.8), most participants were female (*n*=18, 81.8%) and from eight different health professional backgrounds, with nurses being the largest participant group (*n*=10; 45.5%). Practitioners’ length of work within an interprofessional collaborative practice environment varied from 1 to 30 years (*M*=9.1, *SD*=8.6). Students were aged 18 to 40 years (*M*=27.8; *SD*=7.4), all female (*n*=8), and from five different educational backgrounds, with occupational therapy being the largest participant group (*n*=3; 37.5%). Students’ length of study within an interprofessional education and collaborative practice environment varied from 1 to 4 years (*M*=3.0; *SD*=0.9). All students had previous experience working collaboratively in a clinical setting such as in a hospital, aged care or other health centres.

#### Validation study

For factor analysis of validating study data, the adequacy of sample size was determined using the Kaiser-Meyer-Olkin (KMO) test and Bartlett’s Test of Sphericity to evaluate structural validity [38]. In addition, The COSMIN criteria for adequate sample size were followed, which stipulated a sample size >100 and five times the number of items (i.e., 56*5 = 280 participants for each cohort of practitioners and students) [13].

The Australian CPAT was validated by involving healthcare practitioners and students who met the inclusion criteria. The practitioner cohort comprised 152 participants, mainly females (*n*=123, 80.9%). Practitioners’ ages ranged from 20 to 64 years (*M*=37.9; *SD*=10.6). The most common age group was 35–39 years (*n*=39, 25.7%); 58 practitioners were aged under 35 years (38.2%), while 55 practitioners were aged over 39 years (36.2%). Practitioners’ length of service ranged between 1 and 40 years (*M*=13.0; *SD*=10.1), with 6–10 years of service being the highest number of respondents (*n*=34, 22.4%); 48 practitioners with less than six years of service (31.6%); 70 practitioners with more than ten years of service (46.1%). The five most common professional groups were occupational therapists (*n*=33, 21.7%), nurses (*n*=25, 16.4%), speech pathologists (*n*=23, 15.1%), pharmacists (*n*=15, 9.9%) and medicine practitioners (*n*=10, 6.6%). Other professions were dentists, nutritionists, midwives, physiotherapists, podiatrists, psychologists, social workers, ophthalmologists, and public health experts (*n*=46, 30.3%).

The student cohort comprised 247 participants, mainly females (*n*=192, 77.7%). The students’ ages ranged between 18 and 39 years (*M*=24.0; *SD*=5.2). The most common age group was 18–24 years (*n*=174, 70.4%); 73 students were aged over 24 years (29.6%). Students’ length of study within an interprofessional education and collaborative practice environment varied from 1 to 8 years (*M*=2.4; *SD*=1.3), with working experience in health industry such as age and disability care varied from 1 to 4 years (*M*=2.6; *SD*=1.0). The six most common health professional groups were medicine (*n*=37, 15%), nursing (*n*=37, 15%), speech pathology (*n*=28, 11.3%), psychology (*n*=26, 10.5%), occupational therapy (*n*=24, 9.7%), and pharmacy (*n*=24, 9.7%). Other educational backgrounds were dentistry, exercise and sports science, health promotion and sexology, nutrition and dietetics, physiotherapy, and social work (*n*=71, 28.7%).

The total sample size for multi-group analysis met COSMIN very good criteria with a sample >100 in size (total *n*=399) and seven times the number of items (7*56 = 392) [20]. The sample sizes for the individual cohort analyses were in the doubtful range, with sample sizes in both cohorts >100 (Practitioners, *n*=152; Students, *n*=247), but less than five times the number of items tested. However, both datasets qualified for factor analysis, as confirmed by the Kaiser-Meyer-Olkin index (KMO) of 0.900 (practitioners) and 0.962 (students) and the Bartlett test o sphericity with *p*<0.001 (for both datasets).

### Content validity

Content validity reflects the extent to which an instrument’s contents adequately represent the measured construct in the desired cultural setting [13–15]. Content validity can be assessed by asking patients and professionals as the intended users of the instruments being developed [13–15]. Using the COSMIN guide, the first pilot aimed to identify components requiring improvement, whereby participants were presented with the original CPAT and asked to rate each item on the level of *importance (relevance)* and *clarity (comprehensibility)* using a 5-point Likert scale (1 = Strongly Disagree, 2 = Disagree, 3 = Neither agree nor disagree, 4 = Agree, to 5 = Strongly Agree). To explore participants’ opinions further, those who chose *disagree* or *strongly disagree* were directed to open-ended questions that invited them to provide their reasoning and suggest alternative words. Additionally, to assess the *comprehensiveness* of the instrument, participants were asked to share any topics they felt were missing from the instrument. The results of Pilot 1 were then used as the basis for item revisions. To this end, seven items were rewritten to improve clarity. The second pilot was conducted to verify the changes made to the instrument. Agreement scores of >70% for relevance were expected for each item for inclusion.

Qualitative data from the open-ended questions related to participants’ responses to the items’ *clarity (comprehensibility)* and *comprehensiveness* in the pilot studies were analysed using content analysis [39]. The instrument resulting from this two-phased pilot study is hereinafter referred to as the Australian CPAT.

### Internal structure

The Australian CPAT was presented to participants in the validation study. The participants were asked to rate each item on a 1 to 7-point Likert scale (1 = Strongly Disagree, 2 = Mostly disagree, 3 = Somewhat disagree, 4 = Neither agree nor disagree, 5 = Somewhat agree, 6 = Mostly agree, and 7 = Strongly Agree), following the original instrument scale descriptors [21]. Data from validation were used to evaluate COSMIN criteria for instrument general development requirements (e.g., the sample size and target), internal structure (structural validity, internal consistency reliability, measurement invariance) and hypotheses testing.

#### Structural validity

Structural validity measures the level of representativeness of the instrument’s constructs [15]. Three chronological steps of factorial analysis were performed in this study: Exploratory Factor Analysis (EFA), Confirmatory Factor Analysis (CFA), and Multi-Group Confirmatory Factor Analysis (MG-CFA). First, EFA was conducted separately for each group to explore the factorial structure of each group to determine whether or not to maintain the 8-factor 56-item model for the Australian CPAT [21]. The exploratory factor analysis solution may be weak because items can load on any factor [38]. For this reason, the original 8-Factor 56-Item solution was to be maintained whenever possible to the exact factorial numbers and item positioning unless there were strong EFA indications to do otherwise. A structural model informed by the exploratory factor analysis was used as the initial model for CFA. Second, confirmatory factor analysis was carried out separately for each cohort based on the CFA initial model. Relevant CFA results from each cohort (e.g., item estimates, convergent validity, composite reliability, average variance extracted, and model fit indices) were used to determine whether certain factors should be combined or if items should be removed to inform the development of the CFA final model for the Australian CPAT. For invariant measurement, we need to confirm that the two cohorts agreed when jointly analysed using the CFA final model. Therefore, the two cohorts were jointly analysed in the third step of MG-CFA. If MG-CFA shows a good model fit, measurement invariance can be carried out. Measurement invariance involved the analyses of both datasets simultaneously [19, 20].

Data with less than 75% response rate were discarded; missing data at response rates >75% were replaced with mean average values. Data analysis was performed with the Statistical Package for the Social Sciences (SPSS) 26 and Analysis Movement of Structure (AMOS) 24 [40]. This study used COSMIN criteria for good fit indices based on either comparative fit index (CFI), Tucker-Lewis index (TLI) or comparable measure >0.95, **OR** root mean square error of approximation (RMSEA) <0.06, **OR** standard root mean square of the residual (SRMR) <0.08 [15]. This study used these COSMIN indices cut-offs to assess a model fit throughout [15].

#### Internal consistency reliability

Internal consistency refers to the relatedness of the observed constructs and how these constructs are correlated in measuring the same general concept [15]. Depending on the stage of factor analysis, Cronbach *α* and Composite reliability scores were calculated in this study for each subscale to confirm their unidimensionality [15, 41, 42]. A score of 0.7 is acceptable, with a score greater than 0.80 considered high. Scores greater than 0.95 are undesirable, as such high scores suggest item redundancy rather than homogeneity [15]. To measure the convergent validity shared between the construct and its individual indicators, the average variances extracted (AVE) for each factor were also calculated [42]. The AVE is expected to be a minimum of 0.5 or higher [42]. However, if the composite reliability is > 0.6, then an AVE of 0.4 is considered acceptable.

#### Measurement invariances

Assuming that the conformation of the model produced by the multi-group confirmatory factor analysis gave good fit indices, the psychometric evaluation was continued with invariance tests to assess the equivalence of the models when used for cross-group measurement [19]. Invariance tests included configural, metrics and scalars tests. Each level of invariance testing applied constraints that were predicted to cause a decrease in the fit indices [19]. To confirm invariance, differences in comparative fit index (ΔCFI) values were expected to ≤ 0.01 [20]. COSMIN’s criteria for good fit indices were used as cut-offs [15].

Configural invariance tested the model by comparing the structure of the tested groups based on the independently estimated number of latent and observable variables (i.e., testing the model without constraints) [19, 20]. A good model fit indicates that the data passes configural invariance across groups and serves as confirmation to continue testing metrics invariance. Metric invariance was set with each item factor loading constrained equally across groups [19, 20]. The differences in the comparative fit index and fit indices of the metric and configural models were then compared (an index of ≤ 0.01 confirmed metric invariance) [19, 20]. A good model fit indicates that the data passes metric invariance across groups and serves as confirmation to continue testing scalar invariance [19, 20]. Scalar invariance imposes additional constraints to the item factor loading, where the item intercept was equalised between groups [19, 20]. The differences in the comparative fit index of ≤ 0.01 between the metric and scalar models confirmed scalar invariance.

### Hypotheses testing

The nature of the assumptions was identified using the referred model of path analysis [35], whether it is a full or partial mediation. Full mediation is when the assumption results in a significant indirect (mediated) relationship, with a non-significant direct relationship from the antecedent to a consequence factor; whereas partial mediation is when the assumption results in significant effects for both indirect (mediated) and direct (without mediation) relationships [43]. The direct hypotheses (HDir.1 to 8) and indirect hypotheses (HInd.1 to 8) tested for each dataset were as follows:

**Direct paths**:

HDir.1. *Team leadership* has significant direct effects on *General relationship*
HDir.2. *Team leadership* has significant direct effects on *Decision-making and conflict management*.
HDir.3. *Team leadership* has significant direct effects on *Community linkages and coordination of care*.
HDir.4. *Team leadership* has significant direct effects on *Patient involvement*.
HDir.5. *Communication and information exchange* has significant direct effects on *General relationship*.
HDir.6. *Communication and information exchange* has significant direct effects on *Decision-making and conflict management*.
HDir.7. *Communication and information exchange* has significant direct effects on *Community linkages and coordination of care*.
HDir.8. *Communication and information exchange* has significant direct effects on *Patient involvement*.

**Indirect paths**:

HInd.1. The mediators (*Mission, meaning, purpose and goals* and *General roles responsibilities, autonomy*) mediated a significant indirect effect for *Team leadership* on *General relationships*.
HInd.2. The mediators (*Mission, meaning, purpose and goals* and *General roles responsibilities, autonomy*) mediated a significant indirect effect for *Team leadership* on *Decision-making and conflict management*.
HInd.3. The mediators (*Mission, meaning, purpose and goals* and *General roles responsibilities, autonomy*) mediated a significant indirect effect for *Team leadership* on *Community linkages and Coordination of care*.
HInd.4. The mediators (*Mission, meaning, purpose and goals* and *General roles responsibilities, autonomy*) mediated a significant indirect effect for *Team leadership* on *Patient involvement*.
HInd.5. The mediators (*Mission, meaning, purpose and goals* and *General roles responsibilities, autonomy*) mediated a significant indirect effect for *Communication and information exchange* on *General relationships*.
HInd.6. The mediators *(Mission, meaning, purpose and goals* and *General roles responsibilities, autonomy*) mediated a significant indirect effect for *Communication and information exchange* on *Decision-making and conflict management*.
HInd.7. The mediators *(Mission, meaning, purpose and goals* and *General roles responsibilities, autonomy*) mediated a significant indirect effect for *Communication and information exchange* on *Community linkages and coordination of care*.
HInd.8. The mediators *(Mission, meaning, purpose and goals* and *General roles responsibilities, autonomy*) mediated a significant indirect effect for *Communication and information exchange* on *Patient involvement*.

The direct path hypotheses testing were carried out using AMOS (Version 24). Additional estimand [44] was incorporated into AMOS-24 to enable individual indirect path calculations.

## Results

In line with the research objective to provide a quality and psychometrically sound instrument to measure interprofessional education and collaborative practice for both healthcare practitioners and students in Australia, the findings are presented following the COSMIN guidelines for content validity, internal structure (structural validity, internal consistency reliability, and measurement invariance), and hypothesis testing. The procedures are depicted in Fig 1.

### Content validity

All 56 items had agreement scores greater than 70% for *importance (relevance)* of items for inclusion. Concerning *clarity (comprehensibility)*, all items reached >70% agreement. However, seven items had low mean scores and high standard deviation (*M* ≤ 3.5; *SD* >1). Detailed information regarding all item means and standard deviations for the pilot study is presented in the supplementary S1 Table. No participants raised issues regarding the *comprehensiveness* of the instrument.

Participants provided alternative wording for some items, which were then reviewed by the research panel. As a result, seven items were reworded. The comparison of original and modified statements of these items is provided in the supplementary S1 Table. All seven items were retested in the second pilot with similar participants four weeks after the previous pilot. All items tested reached >70% agreement for importance (relevance) and clarity (comprehensibility). Therefore, no further pilot testing was needed. All 56 items were included for validation.

### Structural validity

#### Exploratory factor analysis (EFA)

EFA was performed with dimensional reduction set to maximum likelihood extraction and varimax rotation. Eigenvalues >1 resulted in no items with extraction communalities of < 0.300. The results indicated 12 potential factors in the practitioner cohort and 8 in the student cohort (with a cumulative percentage explaining 73.2% and 71.6% of the variance, respectively).

In both cohorts, five distinct factors were identified with items’ positioning to relevant factors closely matching the original CPAT 8-Factor 56-Item configuration. The stability of these five factors was further confirmed when the number of factors was sequentially reduced. In addition, items that were not correlated with these five factors were also explored separately for the best positioning. The variation of the steps carried out corroborates the possibility of the 8-Factor 56-Item structure as the most suitable model for both cohorts. Furthermore, the internal consistency reliability for each subscale and total instrument exceeded Cronbach’s *α* score of 0.70, and the inter-item correlations were mainly within the expected range of 0.3–0.5. Therefore, the 8-Factor 56-Item solution suggested by the original instrument [21] was used as the initial model for CFA. EFA results regarding items factor loadings and scree plot for each dataset are provided in the S1 File.

#### Confirmatory factor analysis (CFA)

CFA started by analysing the initial model as suggested by the original CPAT. CFA results in the initial model showed 54 items with adequate factor loadings (estimates>0.500), good critical ratio (CR>1.96), and making a significant contribution to its corresponding factor (*p*<0.001 at 95% Confidence interval). Two items, Item 27 (*Physicians assume the ultimate responsibility*) and Item 49 (*Final decision rest with the physician*), were with the lowest factor loadings in both cohorts, with CR<1.96 and *p*>0.05. Detailed information on each item’s regression weights (estimates), critical ratio (CR), and probability as an item that contributes to the relevant factor is provided in Table 1.

**Table 1:** Item estimates, standard error, critical ratio and *p*-value.

Factors	Items	Practitioner				Student			
		Estimate^1^	S.E.^2^	C.R.^3^	***p***	Estimate^1^	S.E.^2^	C.R.^3^	***p***
F1	C1	0.573	0.106	7.153	<0.001	0.734	0.065	12.823	<0.001
	C2	0.465	0.083	5.685	<0.001	0.692	0.068	11.900	<0.001
	C3	0.785	0.090	10.324	<0.001	0.797	0.058	14.340	<0.001
	C4	0.655	0.140	8.313	<0.001	0.668	0.071	11.391	<0.001
	C5	0.834	0.116	11.125	<0.001	0.822	0.066	14.968	<0.001
	C6	0.792	0.079	10.439	<0.001	0.844	0.060	15.543	<0.001
	C7	0.628	0.108	7.932	<0.001	0.764	0.062	13.530	<0.001
	C8	0.778	-	-	-	0.804	-	-	-
F2	C9	0.594	0.127	6.968	<0.001	0.791	0.054	15.510	<0.001
	C10	0.733	0.185	8.549	<0.001	0.849	0.067	17.513	<0.001
	C11	0.408	0.180	4.818	<0.001	0.735	0.067	13.809	<0.001
	C12	0.733	0.147	8.555	<0.001	0.823	0.063	16.572	<0.001
	C13	0.889	0.170	10.268	<0.001	0.840	0.058	17.166	<0.001
	C14	0.709	0.166	8.287	<0.001	0.835	0.062	16.981	<0.001
	C15	0.852	0.164	9.871	<0.001	0.822	0.058	16.523	<0.001
	C16	0.700	-	-	-	0.855	-	-	-
F3	C17	0.771	0.154	7.240	<0.001	0.644	0.127	7.937	<0.001
	C18	0.842	0.141	7.628	<0.001	0.841	0.124	9.273	<0.001
	C19	0.870	0.143	7.768	<0.001	0.869	0.128	9.425	<0.001
	C20	0.875	0.141	7.795	<0.001	0.860	0.128	9.381	<0.001
	C21	0.712	0.144	6.883	<0.001	0.787	0.132	8.952	<0.001
	C22	0.787	0.130	7.334	<0.001	0.857	0.127	9.364	<0.001
	C23	0.831	0.148	7.570	<0.001	0.826	0.138	9.188	<0.001
	C24	0.769	0.132	7.229	<0.001	0.854	0.132	9.345	<0.001
	C25	0.574	-	-	-	0.549	-	-	-
F4	C26	0.627	0.149	6.635	<0.001	0.766	0.080	13.189	<0.001
	C27	**-0.002**	**0.220**	**-0.022**	**0.983**	**0.451**	**0.114**	**7.171**	**<0.001**
	C28	0.588	0.171	6.300	<0.001	0.778	0.079	13.451	<0.001
	C29	0.689	0.150	7.132	<0.001	0.731	0.085	12.447	<0.001
	C30	0.798	0.157	7.936	<0.001	0.801	0.078	13.951	<0.001
	C31	0.374	0.208	4.249	<0.001	0.670	0.091	11.196	<0.001
	C32	0.678	0.130	7.044	<0.001	0.769	0.075	13.245	<0.001
	C33	0.840	0.170	8.219	<0.001	0.793	0.085	13.782	<0.001
	C34	0.675	0.140	7.024	<0.001	0.827	0.074	14.545	<0.001
	C35	0.614	-	-	-	0.782	-	-	-
F5	C36	0.734	0.178	7.673	<0.001	0.756	0.077	12.709	<0.001
	C37	0.899	0.166	8.885	<0.001	0.872	0.072	15.211	<0.001
	C38	0.724	0.146	7.593	<0.001	0.830	0.069	14.281	<0.001
	C39	0.727	0.142	7.611	<0.001	0.820	0.069	14.060	<0.001
	C40	0.842	0.169	8.496	<0.001	0.800	0.079	13.634	<0.001
	C41	0.629	-	-	-	0.773	-	-	-
F6	C42	0.778	0.263	5.603	<0.001	0.799	0.130	10.207	<0.001
	C43	0.794	0.260	5.643	<0.001	0.867	0.127	10.784	<0.001
	C44	0.822	0.263	5.708	<0.001	0.874	0.128	10.838	<0.001
	C45	0.475	-	-	-	0.627	-	-	
F7	C46	0.694	0.089	8.257	<0.001	0.792	0.066	13.368	<0.001
	C47	0.839	0.102	10.003	<0.001	0.829	0.066	14.149	<0.001
	C48	0.828	0.097	9.865	<0.001	0.850	0.066	14.597	<0.001
	C49	**0.080**	**0.137**	**0.947**	**0.344**	**0.525**	**0.079**	**8.296**	**<0.001**
	C50	0.537	0.094	6.373	<0.001	0.720	0.066	11.894	<0.001
	C51	0.717	-	-	-	0.778	-	-	-
F8	C52	0.837	0.148	8.794	<0.001	0.847	0.057	16.794	<0.001
	C53	0.612	0.126	6.751	<0.001	0.778	0.064	14.661	<0.001
	C54	0.838	0.116	8.809	<0.001	0.873	0.053	17.667	<0.001
	C55	0.772	0.149	8.25	<0.001	0.828	0.059	16.179	<0.001
	C56	0.665	-	-	-	0.848	-	-	-

*Note*.

^1^Standardised estimates,

^2^Standar Error,

^3^Critical ratio;

F1 = Mission, Meaning, Purpose, Goals; F2 = General Relationships; F3 = Team Leadership; F4 = General Roles Responsibilities, and Autonomy; F5 = Communication and Information Exchange; F6 = Community Linkages and Coordination of Care; F7 = Decision-Making and Conflict Management; F8 = Patient Involvement.

Model fit indices for the initial 8-Factor 56-items conformation met the COSMIN requirement for a good model fit in both datasets with SRMR values in both cohorts < 0.08 (practitioner dataset: SRMR = 0.074; student dataset: SRMR = 0.058); and CMIN/df were fulfilled with *χ*^2^ (2.113) = 3076.09, CMIN/df = 2.113 for practitioners (cut-off ≤3), and *χ*^2^ (1456) = 3562.50, CMIN/df = 2.447 (cut-off ≤ 3) for students. CFI and RMSEA in both datasets were poor.

Four correlations with estimates > 0.80 in both data sets were identified, which indicated that the corresponding factors were highly correlated. Three correlations were ignored due to conceptual concerns (the corresponding items were not conceptually related), and correlated paired factors were derived from different levels of the construct (see S1 Fig on referenced conceptual framework). Multicollinearity involving F1 *(Mission, meaningful, purpose, and goals)* and F4 *(General roles responsibilities, and autonomy)* was solved by combining the two factors to develop a new domain: *Collective goals and understanding of roles*. Both factors were composed of items with conceptual similarity and framed at the same construct level; both were considered mediators.

**Fig 2:**
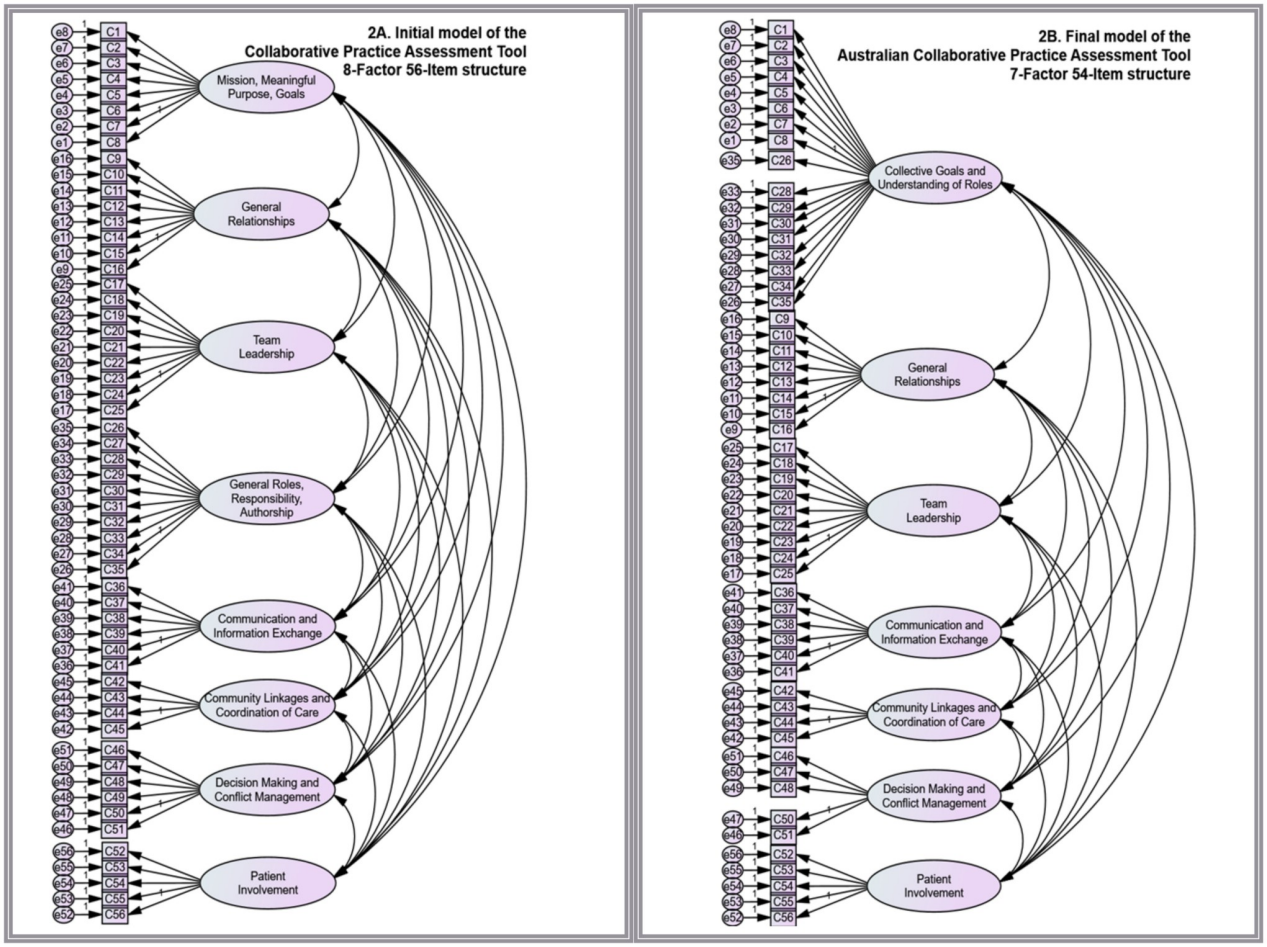
Construct models. A: Initial model of the Collaborative Practice Assessment Tool (8-factor 56-item model). B: Final model of the Australian Collaborative Practice Assessment Tool (7-factor 54-item model).

CFA was rerun with seven factors specifying 54 items (two items were removed, and the factorial structure was reduced as a result of combining two highly related factors). Model fit indices for the final 7-Factor 54-items configuration met the COSMIN requirement for a good model fit in both datasets with SRMR values in both cohorts < 0.08 (practitioner dataset: SRMR = 0.069; student dataset: SRMR = 0.057); and CMIN/df were fulfilled with *χ*^2^ (1.356) = 2768.35, CMIN/df = 2.042 for practitioners (cut-off ≤ 3), and *χ*^2^ (1356) = 3428.00, CMIN/df = 2.528 (cut-off ≤ 3) for students. RMSEA in both datasets improved to be within the acceptable range (practitioner, RMSEA = 0.083; student, RMSEA = 0.078; see Fig 2 for structure comparison). The 7-Factor 54-Items configuration (Fig 2B) was confirmed as the final model for the Australian CPAT and used for further analysis.

#### Multi-group confirmatory factor analysis

MG-CFA was performed simultaneously on two datasets using the 7-Factor 54-Item structure to confirm the model’s fit across two tested groups. The results indicated the achievement of a good model fit with SRMR = 0.058, RMSEA = 0.057, CFI = 0.811, and *χ*^2^ (2.712) = 6197.41, CMIN/df = 2.285. COSMIN’s requirements for good model fit were met and provided justification for invariance testing.

#### Internal consistency reliability

The Australian CPAT demonstrated good internal consistency with high composite reliability (CR) and extracted mean variance (AVE) for all subscales (see Table 2).

**Table 2:** Internal consistency reliability.

Factors	Domains	Practitioner		Student	
		Composite reliability	Average variance extracted	Composite reliability	Average variance extracted
F1	Collective Goals and Understanding of Roles	0.924	0.425	0.953	0.542
F2	General Relationships	0.890	0.513	0.942	0.672
F3	Team Leadership	0.935	0.618	0.938	0.632
F4	Communication and Information Exchange	0.889	0.576	0.918	0.652
F5	Community Linkages and Coordination of Care	0.815	0.534	0.873	0.636
F6	Decision-Making and Conflict Management	0.849	0.534	0.897	0.636
F7	Patient Involvement	0.864	0.564	0.920	0.698

#### Measurement invariance

The invariance tests were carried out on the 7-Factor 54-Item model in the configural, metric and scalar sequence. In the first stage, configural invariance applied no constraints to the model, resulting in a good fit, with SRMR = 0.058, RMSEA = 0.057, CFI = 0.811, and *χ*^2^ (2.712) = 6197.41, CMIN/df = 2.285. These results confirmed configural invariance [19, 20]. The 7-Factor 54-Item solution of the model in terms of the number of factors and the positioning of items on related factors was used for both cohorts.

A good model fit was maintained in the second stage when items’ factor loading was constrained to be equal across groups (SRMR = 0.059, RMSEA = 0.057, CFI = 0.807, and *χ*^2^ (2.759) = 6311.44, CMIN/df = 2.288). The metric variance was confirmed with a CFI difference ≤ 0.01 (a decrease from 0.811 to 0.807) [19, 20]. A good fit was again maintained for the third stage of scalar invariance, in which the loading factor and intercept were constrained to be equal across the groups tested, with SRMR = 0.060, RMSEA = 0.058, CFI = 0.807, and *χ*^2^ (2329) = 6550.58, CMIN/df = 2.329. Scalar variance was confirmed with a CFI difference ≤ 0.01 (a decrease from 0.807 to 0.797) [19, 20]. A summary of the invariance test results is presented in Table 3.

**Table 3:** Full model comparison.

Full Model Comparison	CMIN/df = X^2^	CFI	ΔCFI	SRMR	RMSEA	Invariance
Unconstrained	6197.40/2712 = 2.285	0.811	-	0.058	0.057	Yes
Metric Invariance (Measurement weights)	6311.44/2759 = 2.288	0.807	0.004	0.059	0.057	Yes
Scalar Variance (Measurement intercepts)	6550.58/2813 = 2.329	0.797	0.010	0.060	0.058	Yes

***Note***. CMIN/df = Chi-square minimum discrepancy function, df = Degree of Freedom; CFI = comparative fit index, ΔCFI = differences in CFI, SRMR = standard root mean square of the residual, RMSEA = root mean square error of approximation.

### Hypotheses testing

**Fig 3:**
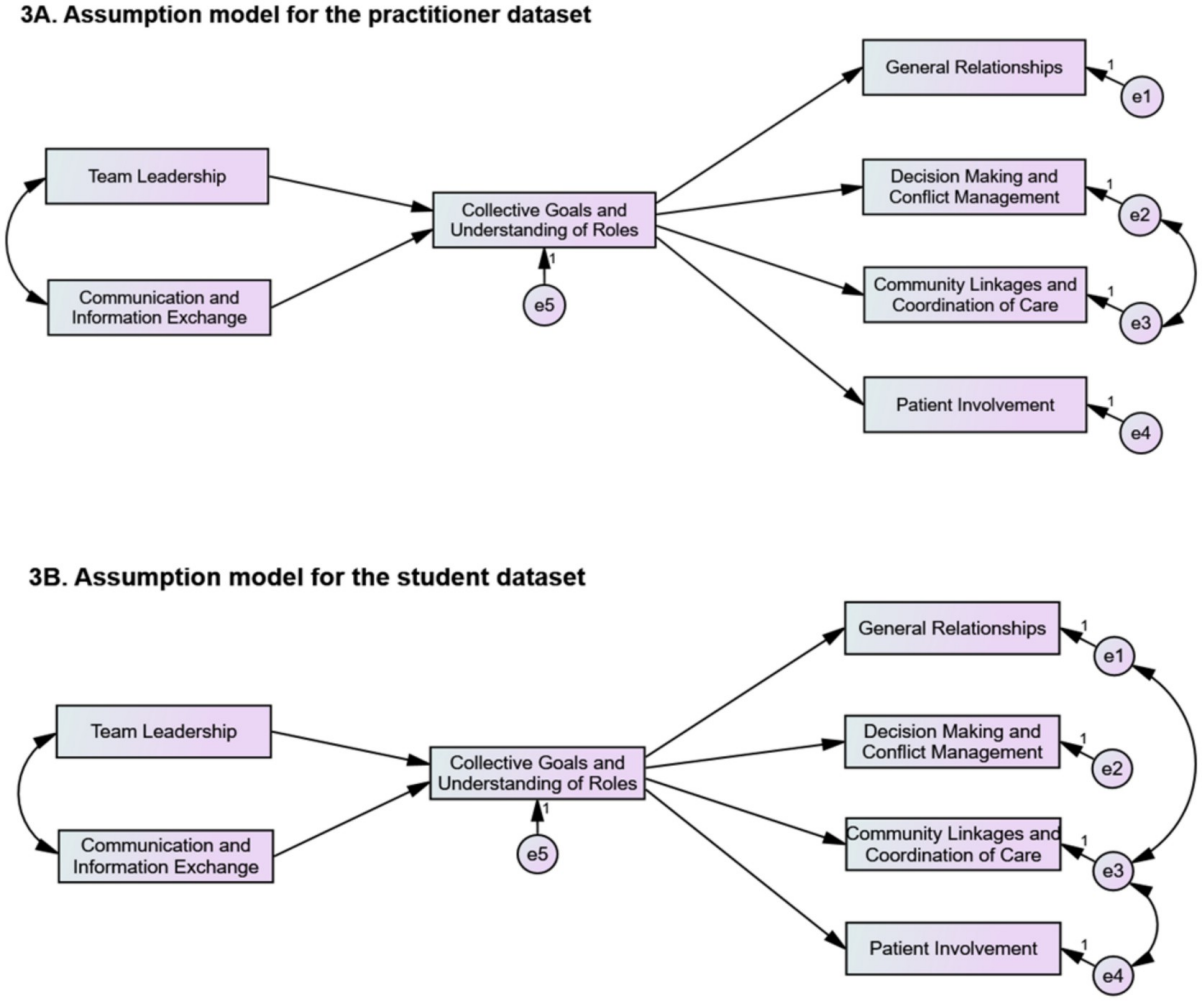
Path analysis of assumptions. 3A. Assumption model for the practitioner dataset. 3B. Assumption model for the student dataset.

The path analysis model used as a reference [35] was adapted to the final factorial solutions obtained from the final CFA to test the hypotheses. Domains *Mission, meaningful, purpose and goals* and *Understanding of roles, responsibilities and autonomy* were combined into a single factor, becoming a unified mediator construct, *Collective goals and understanding of roles*. The proposed path analysis models for the practitioner and student cohorts are shown in Fig 3, respectively.

Some covariance between error terms was generated to improve the fit of the two models. Covariance between error terms in the practitioner dataset significantly improved the SRMR from 0.086 to 0.078 (cut-off < 0.08), and CFI from 0.885 to a more acceptable value of 0.917 with *χ*^2^ (13) = 72.02, CMIN/df = 5.539. The covariances between error terms in the student dataset significantly improved the *χ*^2^ from *χ*^2^ (14) = 114.84, CMIN/df = 8.20 to *χ*^2^ (12) = 82.41, CMIN/df = 6.368 with SRMR = 0.043, and CFI = 0.958. The model fit in both datasets met the requirements for hypotheses testing.

As shown in Fig 3, the indirect effects through the mediation of *Collective goals and understanding of roles* from the antecedent factors of *Team leadership* and *Communication and information exchange* on all consequence factors were all significant in both datasets (HInd.1 to HInd.8 were all accepted). In relation to direct assumptions, the two cohorts have similar rejection related to *Team leadership* to *General relationships* (HDir.1) and *Team leadership* to *Patient involvement* (HDir.4). See Tables 4 and 5 for detailed information on assumption results for both datasets.

**Table 4:** Direct and indirect assumptions for the practitioner.

Relationship	Direct Effect			Indirect Effect			Conclusion
	*β* ^1^	*p*	Hypotheses	*β* ^1^	*p*	Hypotheses	
Team Leadership → Collective Goals and Understanding of Roles → General Relationships	0.016	0.733	HDir.1 **Rejected**	0.148	<0.001	HInd.1 **Accepted**	Full Mediation
Team Leadership → Collective Goals and Understanding of Roles → Decision-Making and Conflict Resolution	0.048	0.147	HDir.2 **Rejected**	0.139	<0.001	HInd.2 **Accepted**	Full Mediation
Team Leadership → Collective Goals and Understanding of Roles → Community Linkages and Coordination of Care	0.085	0.019	HDir.3 **Accepted**	0.064	<0.001	HInd.3 **Accepted**	Partial Mediation
Team Leadership → Collective Goals and Understanding of Roles → Patient Involvement	0.066	0.095	HDir.4 **Rejected**	0.085	<0.001	HInd.4 **Accepted**	Full Mediation
Communication and Information Exchange → Collective Goals and Understanding of Roles → General Relationships	0.298	<0.001	HDir.5 **Accepted**	0.271	<0.001	HInd.5 **Accepted**	Partial Mediation
Communication and Information Exchange → Collective Goals and Understanding of Roles → Decision-Making and Conflict Resolution	0.245	<0.001	HDir.6 **Accepted**	0.289	<0.001	HInd.6 **Accepted**	Partial Mediation
Communication and Information Exchange → Collective Goals and Understanding of Roles → Community Linkages and Coordination of Care	0.208	0.002	HDir.7 **Accepted**	0.132	<0.001	HInd.7 **Accepted**	Partial Mediation
Communication and Information Exchange → Collective Goals and Understanding of Roles → Patient Involvement	0.157	0.029	HDir.8 **Accepted**	0.196	<0.001	HInd.8 **Accepted**	Partial Mediation

*Note*.

^1^Unstandardised estimates; Bootstrap analysis sample = 5000 with a 95% Confidence Interval

**Table 5:** Direct and indirect assumptions for the student.

Relationship	Direct Effect			Indirect Effect			Conclusion
	*β* ^1^	*p*	Hypotheses	*β* ^1^	*p*	Hypotheses	
Team Leadership → Collective Goals and Understanding of Roles → General Relationships	0.096	0.097	HDir.1 **Rejected**	0.336	<0.001	HInd.1 **Accepted**	Full Mediation
Team Leadership → Collective Goals and Understanding of Roles → Decision-Making and Conflict Resolution	0.142	0.002	HDir.2 **Accepted**	0.176	<0.001	HInd.2 **Accepted**	Partial Mediation
Team Leadership → Collective Goals and Understanding of Roles → Community Linkages and Coordination of Care	0.116	0.002	HDir.3 **Accepted**	0.132	<0.001	HInd.3 **Accepted**	Partial Mediation
Team Leadership → Collective Goals and Understanding of Roles → Patient Involvement	-0.024	0.606	HDir.4 **Rejected**	0.221	<0.001	HInd.4 **Accepted**	Full Mediation
Communication and Information Exchange → Collective Goals and Understanding of Roles → General Relationships	0.114	0.163	HDir.5 **Rejected**	0.435	<0.001	HInd.5 **Accepted**	Full Mediation
Communication and Information Exchange → Collective Goals and Understanding of Roles → Decision-Making and Conflict Resolution	0.384	<0.001	HDir.6 **Accepted**	0.160	<0.001	HInd.6 **Accepted**	Partial Mediation
Communication and Information Exchange → Collective Goals and Understanding of Roles → Community Linkages and Coordination of Care	0.165	0.002	HDir.7 **Accepted**	0.170	<0.001	HInd.7 **Accepted**	Partial Mediation
Communication and Information Exchange → Collective Goals and Understanding of Roles → Patient Involvement	0.232	<0.001	HDir.8 **Accepted**	0.179	<0.001	HInd.8 **Accepted**	Partial Mediation

*Note*.

^1^Unstandardised estimates; Bootstrap analysis sample = 5000 with a 95% Confidence Interval

Accepted assumptions reaching 13 out of 16 proposed in both datasets (81.3%), thereby fulfilling the COSMIN requirement of > 75% of the assumptions proposed being accepted [15].

## Discussion

This study aimed to provide a quality and psychometrically sound instrument to measure interprofessional education and collaborative practice outcomes for both healthcare practitioners and students in the Australian setting. The outcomes targeted were related to interprofessional teamwork, communication skills, leadership skills, task and role delineation, conflict management, and involvement with patients and families/communities. The COSMIN (COnsensus-based Standards for the selection of health Measurement INstruments) taxonomy and standards [13–15, 41] were used to guide evaluations of the instrument development requirements of sample target and size, content validity, internal structure (structural validity, internal consistency reliability and measurement invariance), and hypotheses testing.

The 8-Factor 56-Item structure of the original instrument [21], was improved in the Australian CPAT. Based on tiered psychometric evaluation, 7-Factor 54-Item was considered the best factor solution for the Australian CPAT.

Three fundamental changes were made. First, seven items were rewritten, six of which were negatively phrased items (see S1 Table). Negatively worded items were traditionally used to keep participants cognitively alert, to prevent careless answers and serve as correction for agreement bias [45]. However, this finding indicated that negatively worded items introduce unnecessary difficulty for the participants, leading to confusion; similar findings were reported in other psychometric studies [46–48]. The ineffectiveness of items with negative wording to prevent response agreement bias has also been reported, and it can significantly alter the psychometric features of an instrument [48, 49]. The COSMIN content validity check for *comprehensibility (clarity)* was fulfilled after all seven items were positively reworded in the second pilot.

Second, two items were discarded. Item 27 (*Physicians assume the ultimate responsibility*) and Item 49 (*Final decision rest with the physician*) were consistently rejected in the pilot and validation studies, reaffirming participants’ disagreement to include these items. These items did not meet the validity requirements and failed to reflect constructability to the relevant factors [19, 38]. Discarding these items was unavoidable. Notably, these two items were related to physicians’ perceived roles in an interprofessional team. Medical dominance has been widely acknowledged as a barrier to interprofessional collaborative practice [50–53]. This study’s findings substantiate Australian health practitioners’ and students’ disagreement with the concept of medical practitioners as having the highest hierarchical position in decision-making and general patient care.

Third, Factor 1 (*Mission, Meaningful, Purpose, and Goals*) and Factor 4 (*General Roles Responsibilities, and Autonomy*) were combined into one dependent factor, *Collective Goals and Understanding of Roles* (see Fig 2), which reduced the factorial number of Australian CPAT to seven. Statistically, the high estimated correlation between the two factors supported merging the factors. Conceptually, merging was also possible, with both factors being at the same construct level as the referenced model suggests (see S1 Fig) [35]. The seven Australian CPAT subscales each presented as a solid unidimensional domain with good internal consistency reliability and high inter-item correlations, including domain Decision-making and conflict management, which was reported as a weak domain (Cronbach’s score < 0.7) in the original study [21], in the Indonesian [25], and Singaporean validated versions [26]. The fact that the domain of *Decision-making and Conflict Management* is not fully supported in many CPAT studies suggests challenges for decision-making skills in interprofessional teams, which aligns with previous research on the complexity of interprofessional practice [54]. The CPAT validation study in Singapore recommends modification of items related to this domain for use in Asian countries [26]. The Australian CPAT included only five of the six items derived from this domain in the original instrument (item 49 was rejected). Removing this item significantly improved the subscale and total psychometric properties of the Australian CPAT instrument.

However, the composite reliability for Factor 1, *Collective goals and understanding of roles*, exceeded the expected score (Composite reliability = 0.953) in the student dataset. This high score indicated the possibility of redundancy [15, 42]. Two items, Items 5 and 8, identified in both datasets, were associated with a modification index > 20, and were suspected as the source of the redundancy. However, the removal of either or both items was disregarded because the AVE in the student dataset was in the ideal range [42]. Findings from the practitioner dataset supported keeping both items by having composite reliability and AVE values in the acceptable range [42] for the corresponding factor (Factor 1, *Collective goals and understanding of roles*). In addition, removing either item 8 or 5 (or both) or generating a covariance between the two items’ error terms made no significant difference compared to the 7-Factor 54-Item for the MG-CFA model; the RMSEA and SRMS only improved by a maximum of 0.002 points. Convergent validity is met for this measurement model [42].

In line with the targeted objectives, CPAT Australia was configural, metric and scalar invariant in both cohorts. These invariances allow for the use of the instrument in both cohorts as the practitioners and students perceive the meaning of the constructs underlying Australian CPAT in similar ways, and the mean scores of the two cohorts were expected to be comparable when assessed using the suggested model.

The mediation analysis results in this study indicated that practitioners and students perceived *Collective goals and understanding of roles* as an essential mediating variable influencing several antecedents on consequences factors. The findings on the fully mediated assumptions indicated that practitioners and students assumed *Team leadership* can influence a team’s perceptions of their relationships and a team’s encouragement to involve patients in their care, but only if the team was committed to their shared goals and in recognition of their own and others’ roles.

Conversely, as suggested by relationships that were partially mediated (see Tables 4 and 5), practitioners and students were of the view that team leadership and effective communication can directly influence the team’s general relationships, decision-making and conflict management, and community and family empowerment, with or without the need to have collective goals and understanding of roles between team members. The *Collective goals and understanding of roles* were recognised as essential variables in these relationships, however, not as the sole determinant.

All of these results are in accordance with the mediating pattern provided by the suggested model [35]. The results also confirm the notion that practitioners with a greater understanding of their roles and the roles of others are more open to interdependence and working collaboratively to pursue common goals related to patient care [55–57]. The results support findings from previous studies where sharing collective goals and roles understanding are suggested to have a positive influence on dependent variables such as patient satisfaction [58–60], patient involvement [61, 62], team effectiveness [56, 63], and team conflict [64, 65].

The original CPAT was developed in English in Canada. However, cultural differences between Australia and Canada resulted in pilot participants strongly suggesting the rephrasing of seven CPAT items. Two of these were later rejected at the validation stage due to participants’ disagreement with the context being described by the items. Therefore, in alignment with the COSMIN guidelines, cross-cultural validation of an instrument is recommended before using it in different settings or populations.

This study has limitations. Although the number of participants satisfies the COSMIN criteria for very good requirements for multi-group analysis, individual cohort analyses can be improved. The small number of practitioners involved in the study limited the type of analysis that could be conducted (e.g., item response theory cannot be performed). Furthermore, some of the students involved in this study already had experience working in healthcare teams. The students were still in tertiary education and not formally certified health practitioners; however, they had work experience in health centres or clinics such as aged care or disability care. The experiences of students working in healthcare teams provide two sides of a coin for this research. The students’ experience can lead to biased results compared to certified practitioners. However, the experience provides better insight into some aspects of the instrument items, which they would not have been able to respond to had they not experienced contextual interprofessional healthcare teamwork.

## Conclusion

This study presented findings regarding the psychometric evaluation of the Australian CPAT, the procedures and standards of which are based on COSMIN guidelines. Achieving the COSMIN standards confirmed that the evaluation of the psychometric properties of the Australian CPAT informed the quality measure in terms of content validity, structural validity, internal consistency reliability, measurement invariance, and hypotheses testing. Results related to interprofessional teamwork and communication, leadership, task allocation, conflict management, and involving patients and their families/community in care showed the Australian CPAT is invariant for both students and practitioners. The Australian CPAT is, therefore, a quality outcome measure for assessing interprofessional collaborative practice, which can be used for both healthcare practitioners and students in Australia.

## Supporting information

S1 Fig Reference model for hypotheses testing. (TIF)

S1 Table Items modification. (DOCX)

S1 File Additional EFA results. (PDF)

S2 File The Australian Collaborative Practice Assessment Tool. (PDF)

## References

[1] Freeth D. Interprofessional education. Understanding medical education: Evidence, theory and practice. 2013:81–96. doi: 10.1002/14651858.CD002213.pub3 23543515 PMC6513239

[2] World Health Organization, et al. Framework for action on interprofessional education and collaborative practice; 2010. 21174039

[3] Brewer ML, Barr H. Interprofessional education and practice guide no. 8: Team-based interprofessional practice placements. Journal of Interprofessional Care. 2016;30(6):747–53. doi: 10.1080/13561820.2016.1220930 27797631

[4] Brewer M, Flavell H. Facilitating Collaborative Capabilities for Future Work: What Can Be Learnt from Interprofessional Fieldwork in Health. International Journal of Work-Integrated Learning. 2018;19(2):169–80.

[5] Brewer ML, Flavell HL, Jordon J. Interprofessional team-based placements: The importance of space, place, and facilitation. Journal of interprofessional care. 2017;31(4):429–37. doi: 10.1080/13561820.2017.1308318 28467132

[6] Nisbet G, Hendry GD, Rolls G, Field MJ. Interprofessional learning for pre-qualification health care students: An outcomes-based evaluation. Journal of interprofessional care. 2008;22(1):57–68. doi: 10.1080/13561820701722386 18202986

[7] Slater BL, Lawton R, Armitage G, Bibby J, Wright J. Training and action for patient safety: embedding interprofessional education for patient safety within an improvement methodology. Journal of Continuing Education in the Health Professions. 2012;32(2):80–9. doi: 10.1002/chp.21130 22733635

[8] Reeves S, Freeth D. The London training ward: an innovative interprofessional learning initiative. Journal of Interprofessional Care. 2002;16(1):41–52. doi: 10.1080/13561820220104159 11915715

[9] Agency AHPR. Public consultation on the proposed Interprofessional Collaborative Practice Statement of Intent; 2023.

[10] Dunston R, Forman D, Rogers GD, Thistlethwaite J, Yassine T, Hager J, et al. Curriculum renewal for interprofessional education in health: Final report 2014. Commonwealth of Australia, Office for Learning and Teaching; 2014.

[11] Khalili H, Park V, Daulton B, Langlois S, Wetzlmair L, MacMillan KM, et al. Interprofessional Education and Collaborative Practice (IPECP) in Post-COVID Healthcare Education and Practice Transformation Era–Discussion Paper. Joint Publication by InterprofessionalResearch. Global, American Interprofessional Health Collaborative & Canadian Interprofessional Health Collaborative. InterprofessionalResearch. Global; 2022.

[12] Nicol P. Interprofessional education for health professionals in Western Australia: perspectives and activity. Sydney: University of Technology Sydney. 2013.

[13] Mokkink LB, De Vet HC, Prinsen CA, Patrick DL, Alonso J, Bouter LM, et al. COSMIN risk of bias checklist for systematic reviews of patient-reported outcome measures. Quality of Life Research. 2018;27(5):1171–9. doi: 10.1007/s11136-017-1765-4 29260445 PMC5891552

[14] Mokkink LB, Prinsen C, Patrick DL, Alonso J, Bouter L, de Vet HC, et al. COSMIN methodology for systematic reviews of patient-reported outcome measures (PROMs). User manual. 2018;78(1). 10.1007/s11136-018-1798-3 PMC5891568 29435801

[15] Prinsen CAC, Mokkink LB, Bouter LM, Alonso J, Patrick DL, de Vet HCW, et al. COSMIN guideline for systematic reviews of patient-reported outcome measures. Quality of Life Research. 2018;27(5):1147–57. doi: 10.1007/s11136-018-1798-3 29435801 PMC5891568

[16] De Vries DR, Woods S, Fulton L, Jewell G. The validity and reliability of the Interprofessional Socialization and Valuing Scale for therapy professionals. Work. 2016;53(3):621–30. doi: 10.3233/WOR-152224 26835852

[17] King G, Orchard C, Khalili H, Avery L. Refinement of the interprofessional socialization and valuing scale (ISVS-21) and development of 9-item equivalent versions. Journal of Continuing Education in the Health Professions. 2016;36(3):171–7. doi: 10.1097/CEH.0000000000000082 27583993

[18] Ardyansyah BD, Cordier R, Brewer ML, Parsons D. Psychometric evaluation of the culturally adapted interprofessional socialisation and valuing scale (ISVS)-19 for health practitioners and students in Indonesia. Journal of Interprofessional Care. 2024;38(2):283–93. doi: 10.1080/13561820.2023.2285020 38044538

[19] Putnick DL, Bornstein MH. Measurement invariance conventions and reporting: The state of the art and future directions for psychological research. Developmental Review. 2016;41:71–90. doi: 10.1016/j.dr.2016.06.004 27942093 PMC5145197

[20] Cheung GW, Rensvold RB. Evaluating goodness-of-fit indexes for testing measurement invariance. Structural Equation Modeling. 2002;9(2):233–55. doi: 10.1207/S15328007SEM0902_5

[21] Schroder C, Medves J, Paterson M, Byrnes V, Chapman C, O’Riordan A, et al. Development and pilot testing of the collaborative practice assessment tool. Journal of Interprofessional Care. 2011;25(3):189–95. doi: 10.3109/13561820.2010.532620 21182434

[22] Reeves S, Fletcher S, Barr H, Birch I, Boet S, Davies N, et al. A BEME systematic review of the effects of interprofessional education: BEME Guide No. 39. Medical Teacher. 2016;38(7):656–68. doi: 10.3109/0142159X.2016.1173663 27146438

[23] Tomizawa R, Yamano M, Osako M, Misawa T, Hirabayashi N, Oshima N, et al. The development and validation of an interprofessional scale to assess teamwork in mental health settings. Journal of Interprofessional Care. 2014;28(5):485–6. doi: 10.3109/13561820.2014.898623 24625197

[24] Ho CP, Yeh HC, Lee MS, Cheng WC. Reliability and validity of the Taiwanese version of the collaborative practice assessment tool: A pilot study. Tzu Chi Medical Journal. 2023;35(3):267–76. doi: 10.4103/tcmj.tcmj_200_22 37545791 PMC10399841

[25] Yusra RY, Findyartini A, Soemantri D. Healthcare professionals’ perceptions regarding interprofessional collaborative practice in Indonesia. Journal of Interprofessional Education & Practice. 2019;15:24–9. doi: 10.1016/j.xjep.2019.01.005

[26] Quek GSM, Kwan YH, Chan CQH, Phang JK, Low LL. Validation of the collaborative practice assessment tool (CPAT) to assess the degree of inter-professional collaboration (IPC) in a community hospital in Singapore. Journal of Interprofessional Education & Practice. 2022;27:100504. doi: 10.1016/j.xjep.2022.100504

[27] Pfaff KA, Baxter PE, Ploeg J, Jack SM. A mixed methods exploration of the team and organizational factors that may predict new graduate nurse engagement in collaborative practice. Journal of Interprofessional Care. 2014;28(2):142–8. doi: 10.3109/13561820.2013.851072 24195680

[28] Bookey-Bassett S, Markle-Reid M, McKey C, Akhtar-Danesh N. A review of instruments to measure interprofessional collaboration for chronic disease management for community-living older adults. Journal of interprofessional care. 2016;30(2):201–10. doi: 10.3109/13561820.2015.1123233 27026190

[29] Khan AI, Barnsley J, Harris JK, Wodchis WP. Examining the extent and factors associated with interprofessional teamwork in primary care settings. Journal of Interprofessional Care. 2022;36(1):52–63. doi: 10.1080/13561820.2021.1874896 33870838

[30] Paterson ML, Medves J, Dalgarno N, O’Riordan A, Grigg R. The timely open communication for patient safety project. Journal of Research in Interprofessional Practice and Education. 2013;3(1). doi: 10.22230/jripe.2013v3n1a65

[31] Nagelkerk J, Thompson ME, Bouthillier M, Tompkins A, Baer LJ, Trytko J, et al. Improving outcomes in adults with diabetes through an interprofessional collaborative practice program. Journal of Interprofessional Care. 2018;32(1):4–13. doi: 10.1080/13561820.2017.1372395 29111835

[32] Fisher M, Weyant D, Sterrett SE, Ambrose HL. Perceptions of interprofessional collaborative practice and the correlation with patient and family satisfaction scores; 2015.

[33] Findyartini A, Kambey DR, Yusra RY, Timor AB, Khairani CD, Setyorini D, et al. Interprofessional collaborative practice in primary healthcare settings in Indonesia: A mixed-methods study. Journal of Interprofessional Education & Practice. 2019;17:100279. doi: 10.1016/j.xjep.2019.100279

[34] Kang H, Flores-Sandoval C, Law B, Sibbald S. Interdisciplinary health care evaluation instruments: A review of psychometric evidence. Evaluation & the Health Professions. 2022;45(3):223–34. doi: 10.1177/01632787211040859 34409879 PMC9446429

[35] Stutsky BJ, Spence Laschinger H. Development and testing of a conceptual framework for interprofessional collaborative practice. Health and Interprofessional Practice. 2014;2(2):7. doi: 10.7710/2159-1253.1066

[36] Qualtrics. Provo, Utah, USA; version June 2021-May 2022, https://www.qualtrics.com.

[37] Bradby M. Status passage into nursing: another view of the process of socialization into nursing. Journal of Advanced Nursing. 1990;15(10):1220–5. doi: 10.1111/j.1365-2648.1990.tb01715.x 2258530

[38] Williams B, Onsman A, Brown T. Exploratory factor analysis: A five-step guide for novices. Australasian Journal of Paramedicine. 2010;8(3).

[39] Braun V, Clarke V. What can “thematic analysis” offer health and wellbeing researchers? Taylor & Francis; 2014. 10.3402/qhw.v9.26152 PMC4201665 25326092

[40] Corp N. IBM SPSS statistics for windows, version 26. IBM SPSS Corp Armonk, NY; 2017;.

[41] Yoon S, Speyer R, Cordier R, Aunio P, Hakkarainen A. A systematic review evaluating psychometric properties of parent or caregiver report instruments on child maltreatment: Part 2: Internal consistency, reliability, measurement error, structural validity, hypothesis testing, cross-cultural validity, and criterion validity. Trauma, Violence, & Abuse. 2021;22(5):1296–315. 10.1177/1524838020915591 PMC8739544 32270753

[42] Hair JF Jr, Howard MC, Nitzl C. Assessing measurement model quality in PLS-SEM using confirmatory composite analysis. Journal of Business Research. 2020;109:101–10. doi: 10.1016/j.jbusres.2019.11.069

[43] Collier JE. Applied structural equation modeling using AMOS: Basic to advanced techniques. Routledge; 2020.

[44] Gaskin J, Lim J. Indirect effects. AMOS plugin Gaskination’s StatWiki. 2018.

[45] Baumgartner H, Steenkamp JBE. Response styles in marketing research: A cross-national investigation. Journal of marketing research. 2001;38(2):143–56. doi: 10.1509/jmkr.38.2.143.18840

[46] Chyung SY, Barkin JR, Shamsy JA. Evidence-based survey design: The use of negatively worded items in surveys. Performance Improvement. 2018;57(3):16–25. doi: 10.1002/pfi.21749

[47] Johnson JM, Bristow DN, Schneider KC, et al. Did you not understand the question or not? An investigation of negatively worded questions in survey research. Journal of Applied Business Research (JABR). 2004;20(1). doi: 10.19030/jabr.v20i1.2197

[48] Kamoen N, Holleman B, Mak P, Sanders T, Van Den Bergh H. Agree or disagree? Cognitive processes in answering contrastive survey questions. Discourse Processes. 2011;48(5):355–85. doi: 10.1080/0163853X.2011.578910

[49] v Sonderen E, Sanderman R, Coyne JC. Ineffectiveness of reverse wording of questionnaire items: Let’s learn from cows in the rain. PloS one. 2013;8(7):e68967. doi: 10.1371/journal.pone.0068967 23935915 PMC3729568

[50] Bollen A, Harrison R, Aslani P, van Haastregt JC. Factors influencing interprofessional collaboration between community pharmacists and general practitioners—A systematic review. Health & social care in the community. 2019;27(4):e189–212. 30569475 10.1111/hsc.12705

[51] Clarin OA. Strategies to overcome barriers to effective nurse practitioner and physician collaboration. The Journal for Nurse Practitioners. 2007;3(8):538–48. doi: 10.1016/j.nurpra.2007.05.019

[52] Mian O, Koren I, Rukholm E. Nurse practitioners in Ontario primary healthcare: referral patterns and collaboration with other healthcare professionals. Journal of interprofessional care. 2012;26(3):232–9. doi: 10.3109/13561820.2011.650300 22256946

[53] Fejzic J, Emmerton L, Tett S. Towards concordance in healthcare: perspectives of general practitioners, complementary and alternative medicine practitioners and pharmacists in Australia. Journal of clinical pharmacy and therapeutics. 2010;35(3):309–21. doi: 10.1111/j.1365-2710.2009.01093.x 20831532

[54] Xyrichis A, Reeves S, Zwarenstein M. Examining the nature of interprofessional practice: An initial framework validation and creation of the InterProfessional Activity Classification Tool (InterPACT). Journal of interprofessional care. 2018;32(4):416–25. doi: 10.1080/13561820.2017.1408576 29236560

[55] Clark PG. Examining the interface between interprofessional practice and education: Lessons learned from Norway for promoting teamwork. Journal of interprofessional care. 2011;25(1):26–32. doi: 10.3109/13561820.2010.497751 20795829

[56] MacDonald MB, Bally JM, Ferguson LM, L Murray B, Fowler-Kerry SE, Anonson JM. Knowledge of the professional role of others: A key interprofessional competency. Nurse education in practice. 2010;10(4):238–42. doi: 10.1016/j.nepr.2009.11.012 20308019

[57] Atwal A, Caldwell K. Do multidisciplinary integrated care pathways improve interprofessional collaboration? Scandinavian journal of caring sciences. 2002;16(4):360–7. doi: 10.1046/j.1471-6712.2002.00101.x 12445105

[58] Lawrence D, Bryant TK, Nobel TB, Dolansky MA, Singh MK. A comparative evaluation of patient satisfaction outcomes in an interprofessional student-run free clinic. Journal of Interprofessional Care. 2015;29(5):445–50. doi: 10.3109/13561820.2015.1010718 25700220

[59] San Martin-Rodriguez L, D’Amour D, Leduc N. Outcomes of interprofessional collaboration for hospitalized cancer patients. Cancer nursing. 2008;31(2):E18–27. doi: 10.1097/01.NCC.0000305701.99411.ac 18490877

[60] Will KK, Johnson ML, Lamb G. Team-based care and patient satisfaction in the hospital setting: a systematic review. Journal of Patient-Centered Research and Reviews. 2019;6(2):158. doi: 10.17294/2330-0698.1695 31414027 PMC6676761

[61] Spence Laschinger HK, Gilbert S, Smith LM, Leslie K. Towards a comprehensive theory of nurse/patient empowerment: applying Kanter’s empowerment theory to patient care. Journal of nursing management. 2010;18(1):4–13. doi: 10.1111/j.1365-2834.2009.01046.x 20465724

[62] Snyder H, Engström J. The antecedents, forms and consequences of patient involvement: a narrative review of the literature. International Journal of Nursing Studies. 2016;53:351–78. doi: 10.1016/j.ijnurstu.2015.09.008 26602069

[63] Mitchell RJ, Parker V, Giles M. When do interprofessional teams succeed? Investigating the moderating roles of team and professional identity in interprofessional effectiveness. Human relations. 2011;64(10):1321–43. doi: 10.1177/0018726711416872

[64] Brown J, Lewis L, Ellis K, Stewart M, Freeman TR, Kasperski MJ. Conflict on interprofessional primary health care teams–can it be resolved? Journal of interprofessional care. 2011;25(1):4–10. doi: 10.3109/13561820.2010.497750 20795830

[65] Gergerich E, Boland D, Scott MA. Hierarchies in interprofessional training. Journal of interprofessional care. 2019;33(5):528–535. doi: 10.1080/13561820.2018.1538110 30383437

