# Supplementary figures and images for: An evaluation of the psychometric properties of the Australian Collaborative Practice Assessment Tool
Source: PLoS One. 2024 May 9;19(5):e0302834. doi: 10.1371/journal.pone.0302834 (PMC11081231; doi:10.1371/journal.pone.0302834)

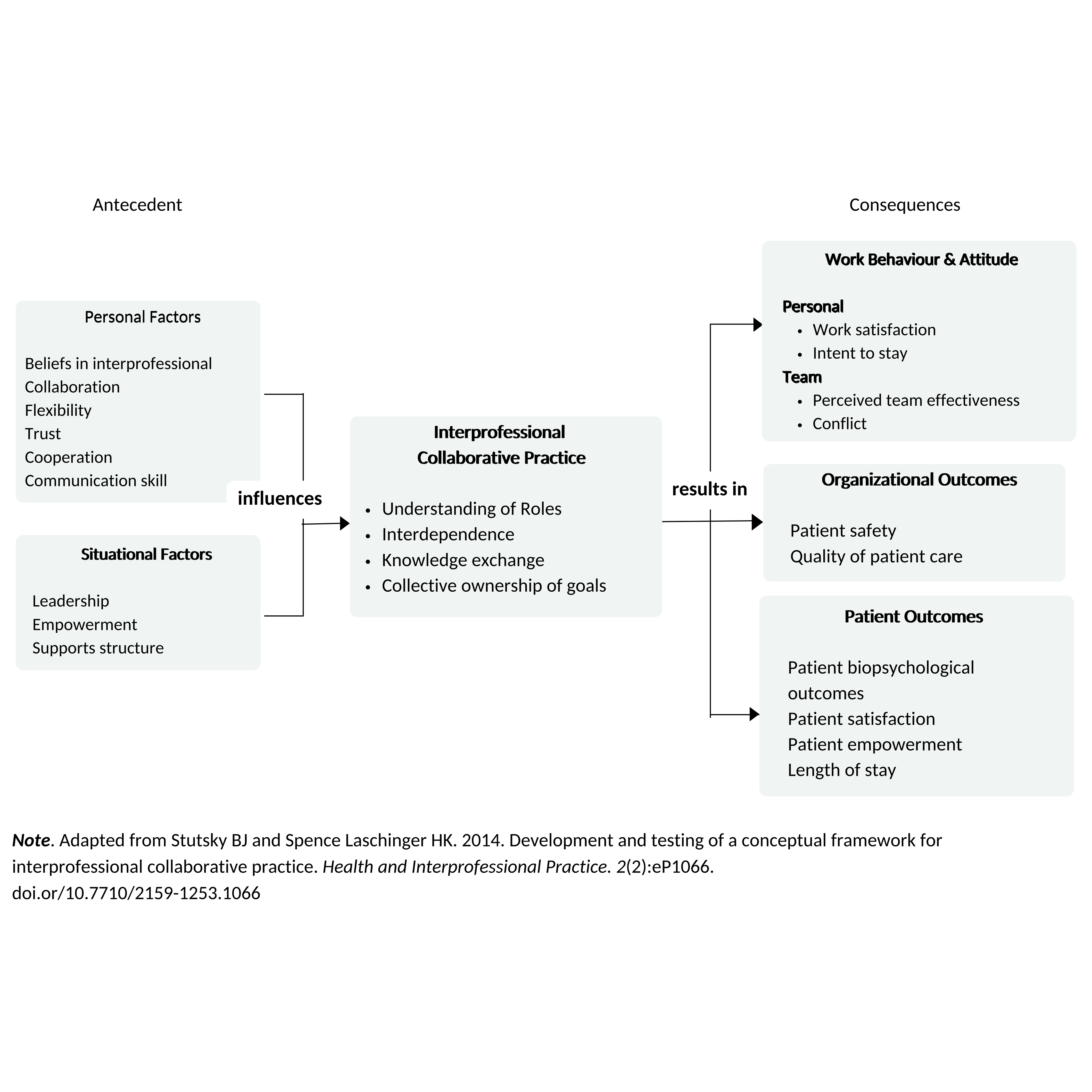

Supplement: S1 Fig — (TIF) [file pone.0302834.s001.tif]
