# Supplementary material for: An evaluation of the psychometric properties of the Australian Collaborative Practice Assessment Tool
Source: PLoS One. 2024 May 9;19(5):e0302834. doi: 10.1371/journal.pone.0302834 (PMC11081231; doi:10.1371/journal.pone.0302834)
Supplement: S1 Table — (DOCX) [file pone.0302834.s002.docx]

**S2 Table. Items modification**

| **Items** | **Original CPAT Items** | **Australian CPAT Items** | | | | | | | | |  |
| --- | --- | --- | --- | --- | --- | --- | --- | --- | --- | --- | --- |
|  |  | **Items** | **Minimum** | | **Maximum** | | **Mean** | | **Std.** | |  |
| CPAT 1 | No changes were made to the item | | | 4 | | 5 | | 4.7 | | 0.5 | |
| CPAT 2 | No changes were made to the item | | | 4 | | 5 | | 4.8 | | 0.4 | |
| CPAT 3 | No changes were made to the item | | | 2 | | 5 | | 4.4 | | 0.9 | |
| CPAT 4 | No changes were made to the item | | | 1 | | 5 | | 4.4 | | 0.9 | |
| CPAT 5 | No changes were made to the item | | | 2 | | 5 | | 4.3 | | 0.9 | |
| CPAT 6 | No changes were made to the item | | | 2 | | 5 | | 4.4 | | 0.8 | |
| CPAT 7 | No changes were made to the item | | | 3 | | 5 | | 4.4 | | 0.7 | |
| CPAT 8 | No changes were made to the item | | | 3 | | 5 | | 4.4 | | 0.6 | |
| CPAT 9 | No changes were made to the item | | | 3 | | 5 | | 4.7 | | 0.5 | |
| CPAT 10 | No changes were made to the item | | | 2 | | 5 | | 4.1 | | 1.0 | |
| CPAT 11 | No changes were made to the item | | | 2 | | 5 | | 4.1 | | 1.0 | |
| CPAT 12 | No changes were made to the item | | | 3 | | 5 | | 4.3 | | 0.8 | |
| CPAT 13 | No changes were made to the item | | | 2 | | 5 | | 4.3 | | 0.9 | |
| CPAT 14 | No changes were made to the item | | | 2 | | 5 | | 4.2 | | 0.9 | |
| CPAT 15 | No changes were made to the item | | | 3 | | 5 | | 4.4 | | 0.7 | |
| CPAT 16 | No changes were made to the item | | | 4 | | 5 | | 4.6 | | 0.5 | |
| CPAT 17 | No changes were made to the item | | | 3 | | 5 | | 4.5 | | 0.7 | |
| CPAT 18 | No changes were made to the item | | | 2 | | 5 | | 4.4 | | 0.8 | |
| CPAT 19 | No changes were made to the item | | | 3 | | 5 | | 4.6 | | 0.6 | |
| **CPAT 20** | Team leadership discourages professionals from taking the initiative to support patient/client care goals. | Our team leader encourages professionals to take the initiative to support patient/client care goals. | 1 | | 5 | | **2.9** | | **1.4** | |  |
| CPAT 21 | No changes were made to the item | | | 2 | | 5 | | 4.4 | | 0.8 | |
| CPAT 22 | No changes were made to the item | | | 3 | | 5 | | 4.5 | | 0.7 | |
| **CPAT 23** | Our team leader is out of touch with team members’ concerns and perceptions. | Our team leader is in touch with the concerns and perceptions of team members. | 1 | | 5 | | **3.1** | | **1.5** | |  |
| CPAT 24 | No changes were made to the item | | | 2 | | 5 | | 4.3 | | 0.8 | |
| CPAT 25 | No changes were made to the item | | | 2 | | 5 | | 4.0 | | 1.0 | |
| CPAT 26 | No changes were made to the item | | | 2 | | 5 | | 4.4 | | 0.8 | |
| **CPAT 27** | Physicians assume the ultimate responsibility for team decisions and outcomes. | In our team, medical doctors assume the ultimate responsibility for team decisions and outcomes. | 1 | | 5 | | **3.4** | | **1.2** | |  |
| CPAT 28 | No changes were made to the item | | | 2 | | 5 | | 3.9 | | 1.1 | |
| CPAT 29 | No changes were made to the item | | | 2 | | 5 | | 4.5 | | 0.8 | |
| CPAT 30 | No changes were made to the item | | | 3 | | 5 | | 4.6 | | 0.6 | |
| CPAT 31 | No changes were made to the item | | | 3 | | 5 | | 4.1 | | 0.7 | |
| CPAT 32 | No changes were made to the item | | | 2 | | 5 | | 4.5 | | 0.7 | |
| CPAT 33 | No changes were made to the item | | | 2 | | 5 | | 4.2 | | 1.0 | |
| CPAT 34 | No changes were made to the item | | | 2 | | 5 | | 4.4 | | 0.8 | |
| **CPAT 35** | Team members feel limited in the degree of autonomy in patient/client care that they can assume. | Team members have a degree of autonomy in patient/client care. | 2 | | 5 | | **3.5** | | **1.1** | |  |
| CPAT 36 | No changes were made to the item | | | 2 | | 5 | | 4.5 | | 0.8 | |
| CPAT 37 | No changes were made to the item | | | 2 | | 5 | | 4.3 | | 0.9 | |
| CPAT 38 | No changes were made to the item | | | 2 | | 5 | | 4.5 | | 0.9 | |
| CPAT 39 | No changes were made to the item | | | 2 | | 5 | | 4.3 | | 0.8 | |
| CPAT 40 | No changes were made to the item | | | 4 | | 5 | | 4.7 | | 0.5 | |
| CPAT 41 | No changes were made to the item | | | 2 | | 5 | | 4.2 | | 1.0 | |
| CPAT 42 | No changes were made to the item | | | 2 | | 5 | | 4.3 | | 0.8 | |
| CPAT 43 | No changes were made to the item | | | 2 | | 5 | | 4.3 | | 0.7 | |
| CPAT 44 | No changes were made to the item | | | 2 | | 5 | | 4.4 | | 0.8 | |
| CPAT 45 | No changes were made to the item | | | 2 | | 5 | | 4.1 | | 1.1 | |
| CPAT 46 | No changes were made to the item | | | 2 | | 5 | | 4.4 | | 0.9 | |
| CPAT 47 | No changes were made to the item | | | 2 | | 5 | | 4.3 | | 1.0 | |
| **CPAT 48** | Disagreements among team members are ignored or avoided. | Disagreements among team members are addressed. | 1 | | 5 | | **2.9** | | **1.7** | |  |
| **CPAT 49** | On our team, the final decision in patient/client care rests with the physician. | The final decision in patient/client care in our team rests with the medical doctor(s). | 2 | | 5 | | **3.5** | | **1.1** | |  |
| **CPAT 50** | In our team, there are problems that regularly need to be solved by someone higher up. | In our team, problems rarely need to be solved by a senior staff member outside of our team. | 1 | | 5 | | **3.2** | | **1.4** | |  |
| CPAT 51 | No changes were made to the item | | | 2 | | 5 | | 4.4 | | 1.0 | |
| CPAT 52 | No changes were made to the item | | | 1 | | 5 | | 4.5 | | 0.9 | |
| CPAT 53 | No changes were made to the item | | | 1 | | 5 | | 4.5 | | 1.0 | |
| CPAT 54 | No changes were made to the item | | | 3 | | 0 | | 4.5 | | 0.7 | |
| CPAT 55 | No changes were made to the item | | | 2 | | 5 | | 4.3 | | 0.9 | |
| CPAT 56 | No changes were made to the item | | | 2 | | 5 | | 4.4 | | 0.8 | |
