# Supplementary material for: An evaluation of the psychometric properties of the Australian Collaborative Practice Assessment Tool
Source: PLoS One. 2024 May 9;19(5):e0302834. doi: 10.1371/journal.pone.0302834 (PMC11081231; doi:10.1371/journal.pone.0302834)

S3 File. Additional EFA Results

A. Items loading factor with Eigenvalue>1 and the scree plot for practitioner dataset

Rotated Factor Matrix[a]

	1	2	3	4	5	6	7	8	9	10	11	12
C20	.820											
C19	.813											
C22	.766											
C18	.756											
C23	.719											
C17	.681											
C21	.668											
C24	.615											
C25	.501											
C51												
C15		.696										
C13		.683										
C26		.570										
C34		.565										
C35		.564										
C30		.528										
C32												
C5			.692									
C6			.603									
C3			.568									
C4			.554									
C8			.532									
C1												
C7												
C38				.691								
C39				.690								
C37				.547								
C40												
C46												
C41												

Total Variance Explained

Factor	Initial Eigenvalues			Extraction Sums of Squared Loadings		
	Total	% of Variance	Cumulative %	Total	% of Variance	Cumulative %
1	21.92	39.14	39.14	3.99	7.12	7.12
2	3.29	5.87	45.01	4.27	7.63	14.75
3	2.45	4.38	49.39	16.66	29.74	44.49
4	2.27	4.06	53.45	2.88	5.14	49.63
5	2.01	3.58	57.04	1.95	3.47	53.11
6	1.75	3.12	60.16	1.58	2.82	55.93
7	1.56	2.79	62.95	1.25	2.24	58.17
8	1.45	2.58	65.53	1.27	2.27	60.44
9	1.29	2.30	67.83	0.94	1.68	62.12
10	1.21	2.16	69.99	1.09	1.95	64.07
11	1.12	2.00	71.99	0.84	1.49	65.56
12	1.08	1.93	73.92	0.80	1.43	66.99
13	0.92	1.65	75.56			
14	0.81	1.44	77.00			
15	0.78	1.39	78.39			

C47												
C48												
C12					.699							
C16					.594							
C9					.564							
C11					.554							
C14					.540							
C10					.501							
C55						.721						
C54						.615						
C52						.548						
C56												
C28							.560					
C36												
C33												
C29												
C42								.776				
C43								.651				
C44								.627				
C27									.988			
C49									.821			
C31												
C2										.655		
C53											.913	
C50												
C45												

Extraction Method: Maximum Likelihood.
Rotation Method: Varimax with Kaiser Normalization.

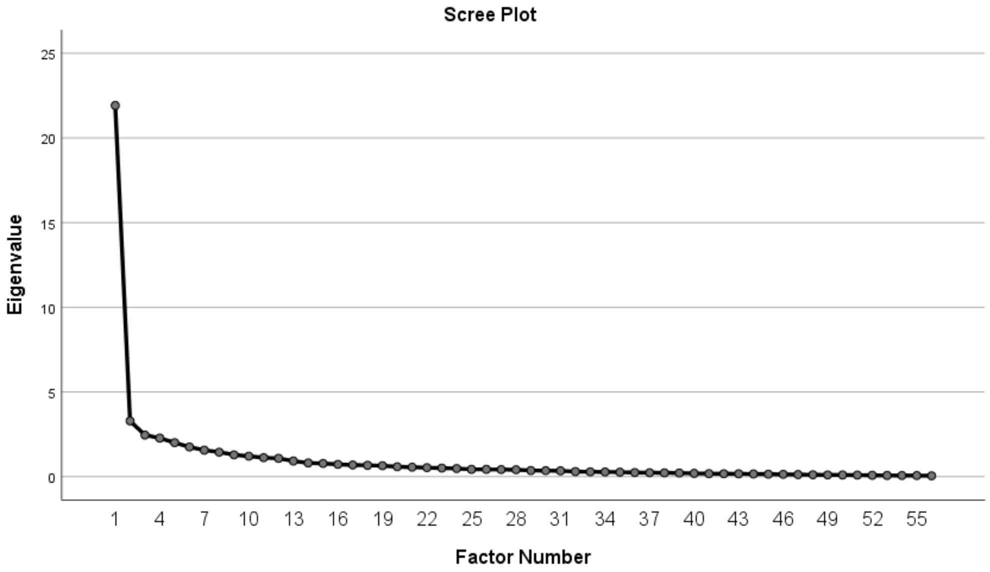

B. Items loading factor with Eigenvalue>1 and the scree plot for student dataset

Rotated Factor Matrix[a]

	1	2	3	4	5	6	7	8
C12	0.776							
C10	0.750							
C13	0.738							
C14	0.737							
C16	0.724							
C8	0.705							
C9	0.684							
C15	0.683							
C5	0.650							
C11	0.648							
C3	0.579							
C7	0.558							
C1	0.555							
C6	0.552							
C26								
C2								
C39								
C17								
C4								
C35								
C47		0.786						
C48		0.778						
C40		0.594						
C51		0.551						
C46								
C33								
C50								
C25								
C41								
C28								
C20			0.726					
C22			0.689					

Total Variance Explained

Factor	Initial Eigenvalues			Extraction Sums of Squared Loadings		
	Total	% of Variance	Cumulative %	Total	% of Variance	Cumulative %
1	28.95	51.70	51.70	28.59	51.06	51.06
2	2.72	4.85	56.55	2.37	4.23	55.29
3	1.99	3.56	60.11	1.69	3.02	58.32
4	1.74	3.10	63.21	1.43	2.55	60.87
5	1.39	2.49	65.70	1.14	2.03	62.90
6	1.17	2.09	67.79	0.88	1.57	64.47
7	1.11	1.98	69.77	0.73	1.30	65.77
8	1.02	1.81	71.58	0.71	1.26	67.03
9	0.96	1.71	73.30			
10	0.90	1.61	74.90			
11	0.81	1.45	76.35			

C19			0.652					
C18			0.624					
C24			0.569					
C23			0.510					
C21			0.508					
C55				0.720				
C54				0.719				
C56				0.694				
C52				0.661				
C53				0.658				
C42					0.773			
C43					0.681			
C44					0.628			
C34						0.524		
C32								
C29								
C31								
C30								
C36								
C27							0.798	
C49							0.749	
C45								
C38								0.503
C37								

Extraction Method: Maximum Likelihood.
Rotation Method: Varimax with Kaiser Normalization.

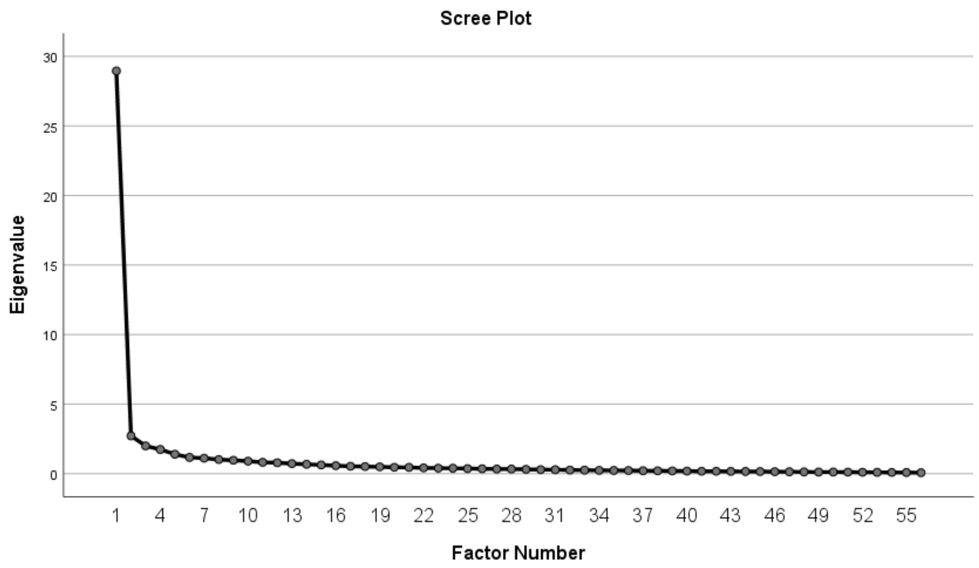

Supplement: S1 File — (PDF) [file pone.0302834.s003.pdf]
