# Supplementary material for: An evaluation of the psychometric properties of the Australian Collaborative Practice Assessment Tool
Source: PLoS One. 2024 May 9;19(5):e0302834. doi: 10.1371/journal.pone.0302834 (PMC11081231; doi:10.1371/journal.pone.0302834)
Supplement: S2 File — (PDF) [file pone.0302834.s004.pdf]

S4 File. The Australian Collaborative Practice Assessment Tool

Collective Goals and Understanding of Roles	Strongly Disagree	Mostly Disagree	Somewhat Disagree	Neither Agree nor Disagree	Somewhat Agree	Mostly Agree	Strongly Agree
1. Our team mission embodies an interprofessional collaborative approach to patient/client care.							
2. Our team's primary purpose is to assist patients/clients in achieving treatment goals.							
3. Our team's goals are clear, useful and appropriate to my practice.							
4. Our team's mission and goals are supported by sufficient resources (skills, funding, time, space).							
5. All team members are committed to collaborative practice.							
6. Members of our team have a good understanding of patient/client care plans and treatment goals.							
7. Patient/client care plans and treatment goals incorporate best practice guidelines from multiple professions.							
8. There is a real desire among team members to work collaboratively.							
26. Team members acknowledge the aspects of care where members of my profession have more skills and expertise.							
28. Team members negotiate the role they want to take in developing and implementing the patient/client care plan.							
29. Team members are held accountable for their work.							
30. It is clear who is responsible for aspects of the patient/client care plan.							
31. Physicians usually ask other team members for opinions about patient/client care.							
32. Team members feel comfortable advocating for the patient/client.							
33. Each team member shares accountability for team decisions and outcomes.							
34. Team members have the responsibility to communicate and provide their expertise in an assertive manner.							
35. Team members have a degree of autonomy in patient/client care.							

**General Relationships**							
9. Respect among team members improves with our ability to work together.							
10. Team members care about one another’s personal well-being.							
11. Socializing together enhances teamwork effectiveness.							
12. It is enjoyable to work with other team members.							
13. Team members respect each other’s roles and expertise.							
14. Working collaboratively keeps most team members enthusiastic and interested in their job.							
15. Team members trust each other’s work and contributions related to patient/client care.							
16. Our team’s level of respect for each other enhances our ability to work together.							
**Team Leadership**							
17. Procedures are in place to identify who will take the lead role in coordinating patient/client care.							
18. Team leadership ensures all professionals needing to participate have a role on the team.							
19. Team leadership assures that roles and responsibilities for patient/client care are clearly defined.							
20. Our team leader encourages professionals to take the initiative to support patient/client care goals.							
21. Team leadership supports interprofessional development opportunities.							
22. Our team leader models, demonstrates and advocates for patient/client-centered best practice.							
23. Our team leader is in touch with the concerns and perceptions of team members.							
24. Our team leader encourages members to practice within their full professional scope.							
25. Our team has a process for peer review.							
**Communication and Information Exchange**							
36. Patients/clients concerns are addressed effectively through regular team meetings and discussion.							
37. Our team has developed effective communication strategies to share patient/client treatment goals and outcomes of care.							

38. Relevant information relating to changes in patient/client status or care plan is reported to the appropriate team member in a timely manner.							
39. I trust the accuracy of information reported among team members.							
40. Our team meetings provide an open, comfortable, safe place to discuss concerns.							
41. The patient/client health record is used effectively by all team members as a communication tool.							
**Community Linkages and Coordination of Care**							
42. Our team has established partnerships with community organizations to support better patient/client outcomes.							
43. Members of our team share information relating to community resources.							
44. Our team has a process to optimize the coordination of patient/client care with community service agencies.							
45. Patient/client appointments are coordinated so they can see multiple providers in a single visit.							
**Decision-making and Conflict Management**							
46. Processes are in place to quickly identify and respond to a problem.							
47. When team members disagree, all points of view are considered before deciding on a solution.							
48. Disagreements among team members are addressed.							
50. In our team, problems rarely need to be solved by a senior staff member outside of our team.							
51. Our team has an established process for conflict management.							
**Patient Involvement**							
52. Team members encourage patients/clients to be active participants in care decisions.							
53. Team members meet face-to-face with patients/clients cared for by the team.							
54. Information relevant to health care planning is shared with the patient/client.							
55. The patient/client is considered a member of their healthcare team.							
56. The patient's/client's family and supports are included in care planning, at the patient's request.							
